# Development of a *Limosilactobacillus reuteri* therapeutic delivery platform with reduced colonization potential

**DOI:** 10.1128/aem.00312-24

**Published:** 2024-10-31

**Authors:** Laura M. Alexander, Saima Khalid, Gina M. Gallego-Lopez, Theresa J. Astmann, Jee-Hwan Oh, Mark Heggen, Phil Huss, Renee Fisher, Amitava Mukherjee, Srivatsan Raman, In Young Choi, Morgan N. Smith, Claude J. Rogers, Michael W. Epperly, Laura J. Knoll, Joel S. Greenberger, Jan-Peter van Pijkeren

**Affiliations:** 1 Department of Food Science, University of Wisconsin-Madison, Madison, Wisconsin, USA; 2 Department of Medical Microbiology and Immunology, University of Wisconsin-Madison, Madison, Wisconsin, USA; 3 Morgridge Institute for Research, Madison, Wisconsin, USA; 4 Department of Biochemistry, University of Wisconsin-Madison, Madison, Wisconsin, USA; 5 Department of Radiation Oncology, UPMC Hillman Cancer Center, Pittsburgh, Pennsylvania, USA; 6 ChromoLogic, LLC, Monrovia, California, USA; Washington University in St. Louis, St. Louis, Missouri, USA

**Keywords:** biotherapeutic, probiotic, adhesins, colonization, engineered probiotic

## Abstract

Bacterial biotherapeutic delivery vehicles have the potential to treat a variety of diseases. This approach obviates the need to purify the recombinant effector molecule, allows delivery of therapeutics *in situ* via oral or intranasal administration, and protects the effector molecule during gastrointestinal transit. Lactic acid bacteria have been broadly developed as therapeutic delivery vehicles though risks associated with the colonization of a genetically modified microorganism have so-far not been addressed. Here, we present an engineered *Limosilactobacillus reuteri* strain with reduced colonization potential. We applied a dual-recombineering scheme for efficient barcoding and generated mutants in genes encoding five previously characterized and four uncharacterized putative adhesins. Compared with the wild type, none of the mutants were reduced in their ability to survive gastrointestinal transit in mice. CmbA was identified as a key protein in *L. reuteri* adhesion to HT-29 and enteroid cells. The nonuple mutant, a single strain with all nine genes encoding adhesins inactivated, had reduced capacity to adhere to enteroid monolayers. The nonuple mutant producing murine IFN-β was equally effective as its wild-type counterpart in mitigating radiation toxicity in mice. Thus, this work established a novel therapeutic delivery platform that lays a foundation for its application in other microbial therapeutic delivery candidates and furthers the progress of the *L. reuteri* delivery system towards human use.

**IMPORTANCE:**

One major advantage to leverage gut microbes that have co-evolved with the vertebrate host is that evolution already has taken care of the difficult task to optimize survival within a complex ecosystem. The availability of the ecological niche will support colonization. However, long-term colonization of a recombinant microbe may not be desirable. Therefore, strategies need to be developed to overcome this potential safety concern. In this work, we developed a single strain in which we inactivated the encoding sortase, and eight genes encoding characterized/putative adhesins. Each individual mutant was characterized for growth and adhesion to epithelial cells. On enteroid cells, the nonuple mutant has a reduced adhesion potential compared with the wild-type strain. In a model of total-body irradiation, the nonuple strain engineered to release murine interferon-β performed comparable to a derivative of the wild-type strain that releases interferon-β. This work is an important step toward the application of recombinant *L. reuteri* in humans.

## INTRODUCTION

Bacteria engineered as therapeutic delivery vehicles are poised to become valuable tools for the future of personalized medicine. Bacteria can be engineered to produce recombinant effector molecules that would otherwise be difficult to manufacture and administer. Serving as both the production factory and delivery system of effector molecules, recombinant bacteria are a powerful chassis to deliver therapeutics following oral or intranasal administration. Several groups have engineered bacteria as delivery vehicles that demonstrated efficacy in various *in vivo* models, and some have recently advanced to human clinical trials (1–5).

An unresolved disadvantage posed by using recombinant bacteria as therapeutic delivery vehicles is the colonization risk. Although there are typically no safety concerns with the (probiotic) chassis that are used to deliver therapeutics, long-term colonization should be avoided to limit the delivery of high doses of certain therapeutics, which can have deleterious side effects. Excessive levels of therapeutic IL-22, for example, correlate with the development of psoriasis and the priming and proliferation of tumors (6–9). Several strategies have been developed to prevent proliferation in the host or the external environment (10). Biocontainment systems developed in the synthetic biology industry include synthetic auxotrophy and both ‘Deadman’ and ‘Passcode’ kill switches (11–13). However, these systems only address the replication ability of recombinant microbes, not their ability to stimulate and interact with host tissue. Another method to eliminate microbes from complex communities is antibiotic treatment, which by itself also is likely to cause negative side effects by disrupting the resident gut microbiota (14). In all of these approaches, however, adhesin proteins on the microbial cell surface continue to allow binding to the host epithelia or mucus. Here, we describe a strategy that reduces the colonization potential of biotherapeutic delivery vehicles by inactivating genes encoding adhesins.

*Limosilactobacillus reuteri* is a gut symbiont species that has evolved to thrive in a large number of vertebrates (15–20), including humans (21). The ability to survive gastrointestinal transit makes select strains exciting candidates to be developed as microbial delivery vehicles of therapeutics in the gut. In particular, human-derived *L. reuteri* strains are exciting candidates due to the available genetic tools (22–25), and its multiple probiotic (*i.e*., health-promoting) characteristics (26–30). Others, and our group, have exploited *L. reuteri* VPL1014 as a chassis to secrete therapeutic molecules (31, 32). More recently, we developed a lysis-based approach to deliver therapeutics (33). Specifically, *L. reuteri* encodes two prophages that are activated by molecules encountered during GI transit, such as short-chain fatty acids (SCFAs), leading to cell lysis (34). We demonstrated that delivery of interleukin-22 following phage-mediated lysis decreases indicators of liver disease in a model of alcohol binge-fed mice (35), while phage-mediated release of interleukin-22 or interferon-β increased the survival of mice exposed to body irradiation (36–39). Now that we have established the potential of *L. reuteri* as a therapeutic delivery vehicle, our goal iss to develop a strain that has reduced colonization potential to bring *L. reuteri* a step closer to the clinic.

**Fig 1:**
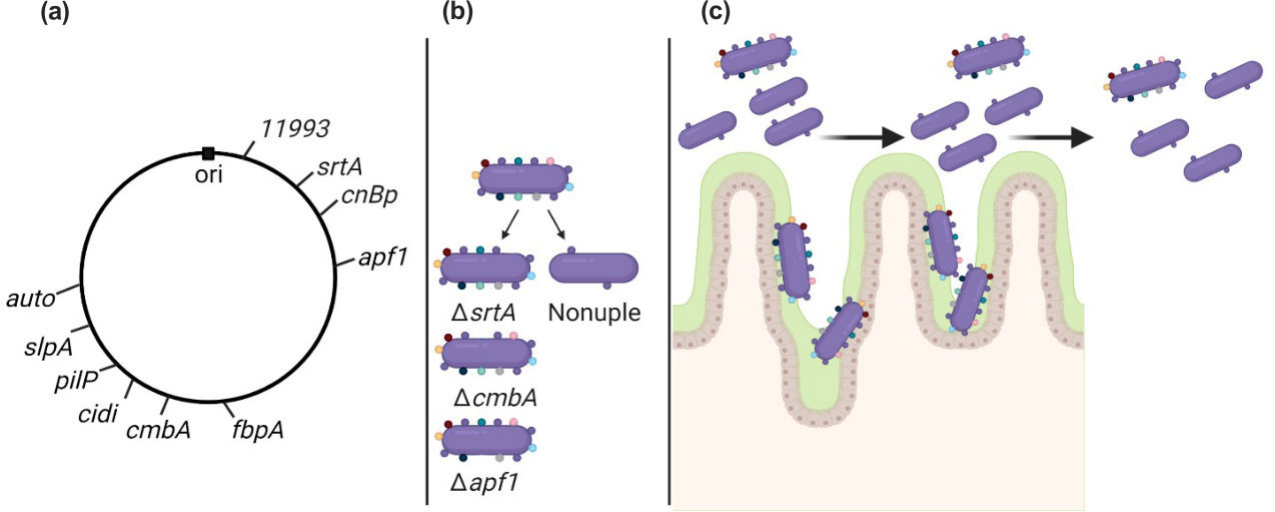
Conceptual overview of a strain with reduced colonization potential. (**a**) The locations of 10 putative adhesion proteins in the *L. reuteri* VPL1014 genome are indicated. (**b**) In this study, we mutated each putative adhesion mutant individually and sequentially to yield a nonuple mutant. Depicted here are graphical examples of single mutants and the nonuple mutant. (**c**) We hypothesize that the nonuple mutant, lacking multiple adhesion proteins, will have reduced adherence to human intestinal cells. In this graphic, wild-type *L. reuteri* can adhere to mucus or epithelial cells, while the nonuple mutant is unable to adhere.

Microbes encode a variety of adhesins that facilitate their association with host cells in the gut. Not only are these interactions important to colonize and persist, but bacterial adhesion also drives modulation of the host immune system (40–42). In Gram-positive bacteria—both pathogenic and probiotics—sortase-dependent proteins (SDPs) represent an important group of surface-associated proteins that impact the adhesion and nutrient acquisition (43). Hallmark features of SDPs include an N-terminal signal peptide for surface localization, a C-terminal region that includes a cell wall-anchoring motif (*e.g*., LPxTG), a transmembrane helix, and a positively charged tail that prevent the protein to be released during processing (44). SDPs are processed by one or more enzymes called sortases. In most lactobacilli, including *L. reuteri*, a single sortase enzyme (SrtA) recognizes the C-terminal LPxTG motif and covalently couples SDPs to the cell wall (40, 43, 45–48). Jensen *et al*. previously identified and tested four *L. reuteri* SDPs for their role in adhesion to human colon cancer cells (Caco-2), which we also included in this study (49). Other, non-SDP adhesins are classified in Gram-positive bacteria based on their specific interactions with host cells and extracellular components, such as fibronectin-binding protein (FbpA) and collagen-binding protein (CnBp) (50, 51). Cell structures, such as S-layer proteins (SlpAs) (52), pili (PilP), flagella, fimbriae, and mucus-binding proteins (Mub and MapA) (53) can also interact with host cells and mucus (54). Finally, surface-associated proteins annotated as autolysins, which contain a cell-wall anchor motif made up of repeated GW modules, play a role in microbe–host interactions by binding heparan sulfate of host cell surface heparan sulfate proteoglycans (55, 56). In addition, aggregation-promoting factors (Apf) (57, 58) play a role in adhesion by facilitating interactions with collagen, fibronectin, and mucus (59). *L. reuteri* VPL1014 lacks proteins and structures homologous to Mub, MapA, pili, and flagella, but it putatively encodes uncharacterized proteins homologous to SlpA, FbpA, autolysin, Apf, and CnBp. The genes encoding these adhesin homologs, along with SDPs, are ideal targets for inactivation to reduce the ability to colonize, persist, or act as an immunomodulator in the human gut (Fig. 1).

Here, we developed a dual-recombineering method that introduces unique tags (barcodes) along with mutations to inactivate genes encoding putative adhesins in *L. reuteri*. With this tool, we targeted nine genes encoding putative adhesins, and *srtA* in *L. reuteri* VPL1014 to evaluate their cumulative role in adhesion. Four targets encoding SDPs were previously characterized (49), and five targets encoding uncharacterized protein homologs are not SDPs but have roles in Gram-positive adhesion to mucins and epithelial cells (50–52, 55, 57, 58, 60). Following the characterization of each of the putative adhesins, we developed and characterized a single strain in which we inactivated nine genes (we excluded one mutation that conferred a growth defect). Functional characterization of this nonuple mutant revealed significantly reduced adhesive ability to human enteroid cells with no reduction in intestinal survival in mice, yet the nonuple strain was cleared faster from the mouse intestinal tract compared with the wild type. Importantly, the therapeutic potential of the nonuple mutant was comparable to that of the wild-type *L. reuteri*. We expect that this novel reduced colonization potential method can be applied to other bacteria engineered for therapeutic delivery, and further advances *L. reuteri* towards implementation in the clinic as a biotherapeutic delivery vehicle.

## RESULTS

To design a mutant strain with reduced adhesive ability, we started our studies by targeting genes encoding sortase-dependent proteins (SDPs) for inactivation. Here, we build on the findings from Jensen *et al*. who identified a single sortase gene and eight additional genes encoding sortase-dependent proteins (SDPs). For this study, we excluded pseudogenes (LAR_1193–1192 and LAR_0089) and genes that are unlikely to play a role in adhesion, which are LAR_0813 and LAR_0903. LAR_0903 has low homology (28% sequence identity) to YggS, a protein important for Vitamin B6 metabolism (61, 62), whereas LAR_0813 is annotated as an amidase.

We used the genome of *L. reuteri* JCM1112 as a reference because it has a completed and closed genome sequence available and is nearly identical to strain *L. reuteri* VPL1014 (63). We searched the JCM1112 genome for functionally characterized protein homologs that have been documented to play a role in adhesion in other lactobacilli. Our analyses revealed five genes: aggregation-promoting factor (*apf1*) (57, 58), fibronectin-binding protein (*fbpA*) (60), surface layer protein (*slpA*) (52), collagen-binding protein (*cnBp*) (50, 51), and autolysin (55) (Table 1). The combined review of the literature along with the *in silico* analysis yielded a total of 10 genes with a putative role in cell and/or mucus attachment. (Table 1).

**TABLE 1:** *In silico* analysis of putative adhesion proteins in *L. reuteri*

Gene target	Locus^*a*^	Rational^*b*^	Predicted SP (Y/N) and YSIRK cleavage site^*c*^ [Y/N]	Hydrophobic CTD^*d*^ region (Y/N)	Sortase dependence(Y/N)	Repeat region (Y/N)	Gap	Identity (%)	Characteristics	Reference
*srtA*	LAR_0227	Sortase	N[N]	Y	n/a^*e*^	N	0	100	Inactivation of *srtA* reduced adhesion to Caco-2 cells	(49)
*cmbA*	LAR_0958	SDP	Y[Y]	Y	Y	Y	0	100	Inactivation of *cmbA* reduced adhesion to Caco-2 cells	(49)
*cnbP*	LAR_0284	Putative collagen-binding protein	Y[N]	Y	N	Y	0	98	*L. reuteri* Pg4 collagen-binding protein (ADN22849.1) Periplasmic binding region	(50)
*fbpA*	LAR_0878	Putative fibronectin-binding protein	N[n/a]	N	N	Y	6/557	55	*L. paracasei* BL23 FbpA, CAQ66743.1)	(60)
*slpA*	LAR_1193	Putative surface layer protein	Y[Y]	Y	N	Y	78/386	62.69	*L. acidophilus* surface layer protein (AJP46713.1)	(52)
*apf1*	LAR_0410	Apf-like domain	Y[N]	Y	N	Y	1/101	78	*L. gasseri* Apf1 (AAO86515.1)	(57)
autolysin	LAR_1284	Putative autolysin	Y[N]	N	N	Y	6/183	44	*L. monocytogenes* autolysin (NP_466081.1) Evidence of role in adhesion in *S*. *aureus*, *L. monocytogenes* and *L. acidophilus*	(55)
*11993*	LAR_0044	Putative SDP	Y[N]	Y	Y	Y	0	100	Inactivation did not result in adherence defect to Caco-2 cells by *L. reuteri*	(49)
ci-phospho-diesterase	LAR_0983	Putative SDP	Y[N]	Y	Y	Y	0	100	Inactivation did not result in adherence defect to Caco-2 cells by *L. reuteri*	(49)
*pilP*	LAR_0989	Putative SDP	Y[N]	Y	Y	Y	0	100	PilP and Rib regions. Inactivation did not result in adherence defect to Caco-2 cells by *L. reuteri*	(49)

^*a*^ Loci are based on the *L. reuteri* reference genome *L. reuteri* JCM1112, and can be accessed at http://www.ncbi.nlm.nih.gov.

^*b*^ SDP, sortase-dependent protein; SDPs were indicated by the presence of: LPxTG motif identified through manual search, YSIRK-G/S (pfam04650) [Y] or non-YSIRK signal sequence [N]), and cell wall anchor domains (TIFR01167).

^*c*^ SP, signal peptide; presence of signal peptide and cleavage site was determined by SignalP-5.0 (64). Protein motifs and domains were identified by Interpro 83.0 and searched for with BLASTP at the National Center for Biotechnology Information website (http://www.ncbi.nlm.nih.gov) (Blum *et al* 2020).

^*d*^ CTD, C-terminal domain.

^*e*^ n/a, not available.

### Development of a mutant library tagging method

**Fig 2:**
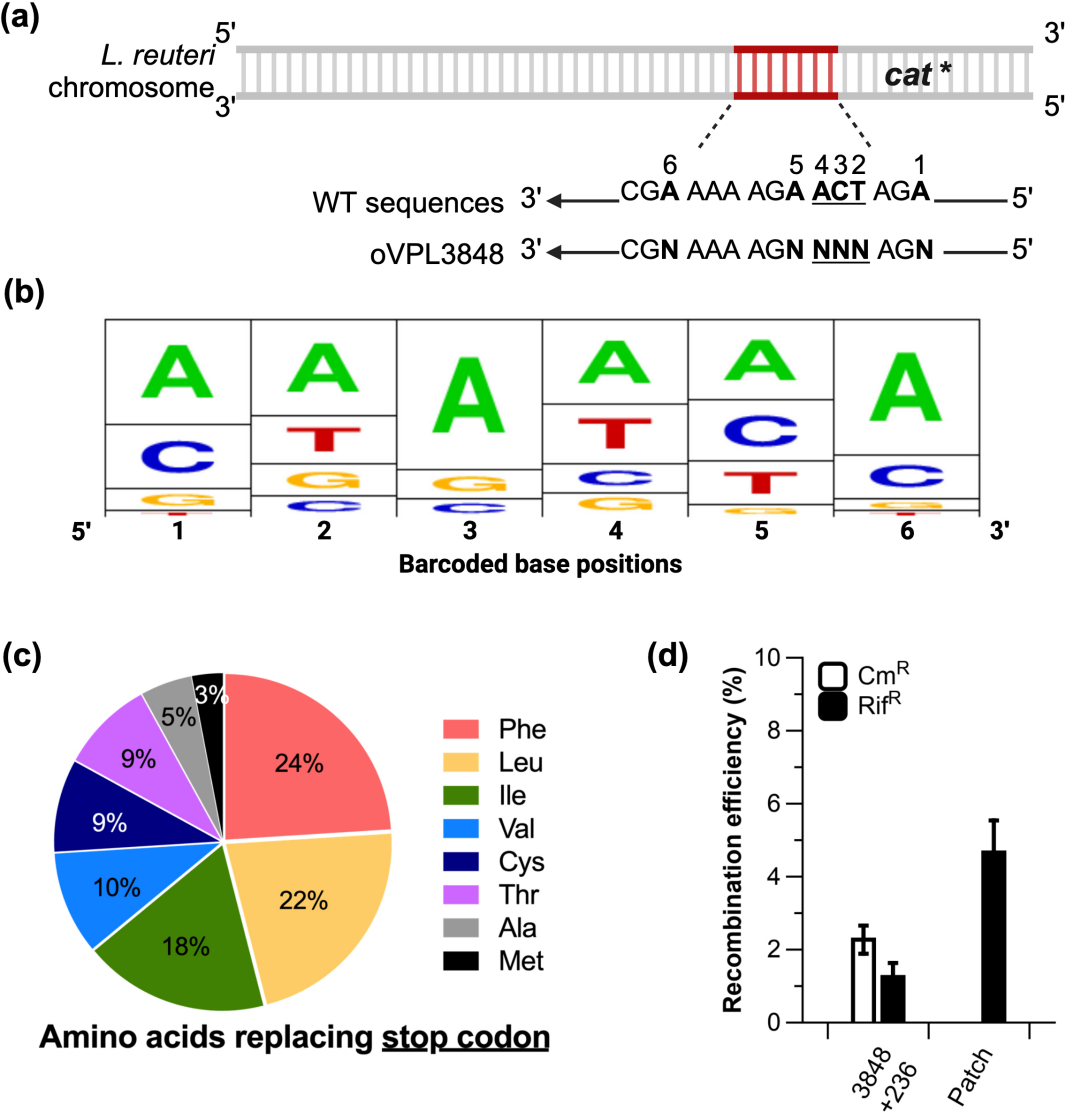
Mutant barcoding scheme and dual-recombineering efficiencies. (**a**) *L. reuteri* VPL4011 contains an insertion of an inactivated chloramphenicol acetyltransferase gene (cat*) coded on the negative strand. Incorporation of oVPL3848, which contains a series of degenerate bases (“N’s”) at the stop codon position (underlined) and at adjacent wobble bases, restores the *cat* gene while barcoding the strain with random bases. (**b**) A sequence logo generated from the sequencing results of 96 barcodes in VPL4011 transformed with oVPL3848. Positions displayed correspond to the barcoded bases (“N’s”) in oVPL3848. Positions 1, 5, and 6 are the wobble base positions, and positions 2–4 are at the location of the replaced stop codon. (**c**) Distribution of amino acids that restored cat* in VPL4011 following transformation with oVPL3848. (**d**) Recombination efficiencies of VPL4011 dual-transformed with oVPL236 (236) and oVPL3848 (3848). Rifampicin-resistant colonies out of 100 patched (patch) colonies from agar plates supplemented with chloramphenicol to agar plates supplemented with rifampicin. The results shown are average values derived from three independent experiments ± standard error of the mean.

To tag recombinant strains, we developed a barcoding system. A chromosomal barcoding system in a gut symbiont will open the door to multiplex the functional characterization of user-defined recombinants. To accomplish this, we first designed a barcoding target. Here, we chose to integrate in the *L. reuteri* chromosome a derivative of the gene encoding chloramphenicol resistance (*cat*) that contained an in-frame stop codon to yield strain VPL4011. To generate barcodes in the chromosome, we applied single-stranded DNA recombineering using a degenerate oligonucleotide (oVPL3848) that, when incorporated, repairs the stop codon in the *cat* gene and generates mutations at wobble base positions creating unique tags (Fig. 2a). To map the distribution of mutations generated, we sequenced the *cat* gene of 96 chloramphenicol-resistant colonies; we observed 81 unique barcodes and 15 repeated barcodes (Fig. 2b). Adenines were overrepresented at positions 1 and 6 (which are the original bases at those positions), and position 3, which is within the codon that replaces the stop codon. Bases at positions 2 and 4 are evenly distributed, while guanine is underrepresented at position 5. Notably, only eight of 20 amino acids were represented in the 96 transformants as replacements for the stop codon (Fig. 2c). Thus, without the use of a purified recombineering oligonucleotide and optimization studies, our approach provides a robust means to create nearly 100 unique chromosomal tags in a single step.

We chose this barcoding method because it enabled us to screen for recombinants within a pool of cells that have successfully undergone a recombineering event, *i.e*., are chloramphenicol resistant. We hypothesized that this approach would recover recombinants at a higher frequency compared with a conventional recombineering approach that does not employ antibiotic selection. To test this, we simultaneously dual-transformed strain VPL4011 with oligonucleotides oVPL3848 and oVPL236, which—upon successful incorporation—repair the stop codon in the *cat* gene and generate mutations in the gene encoding RNA polymerase B (*rpoB*), respectively. The mutations in *rpoB* render the cells resistant to rifampicin (22). Plating of the dual-transformation on MRS agar supplemented with chloramphenicol or rifampicin revealed recombination efficiencies of 2.47 ± 0.39% and 1.25 ± 0.39**%** relative to the total CFU, respectively (Fig. 2d). To determine the dual-recombineering efficiency, we patch-plated 100 dual-transformed colonies from plates containing chloramphenicol onto plates containing rifampicin, which revealed that six of 100 colonies (6%) were resistant to both antibiotics. Thus, we recovered approximately 5-fold more recombinants when we screened a pool of cells that had successfully undergone a recombineering event. This placed us in the position to apply this approach to inactivate genes putatively encoding adhesins.

### Single-mutant library construction and growth characterization

**Fig 3:**
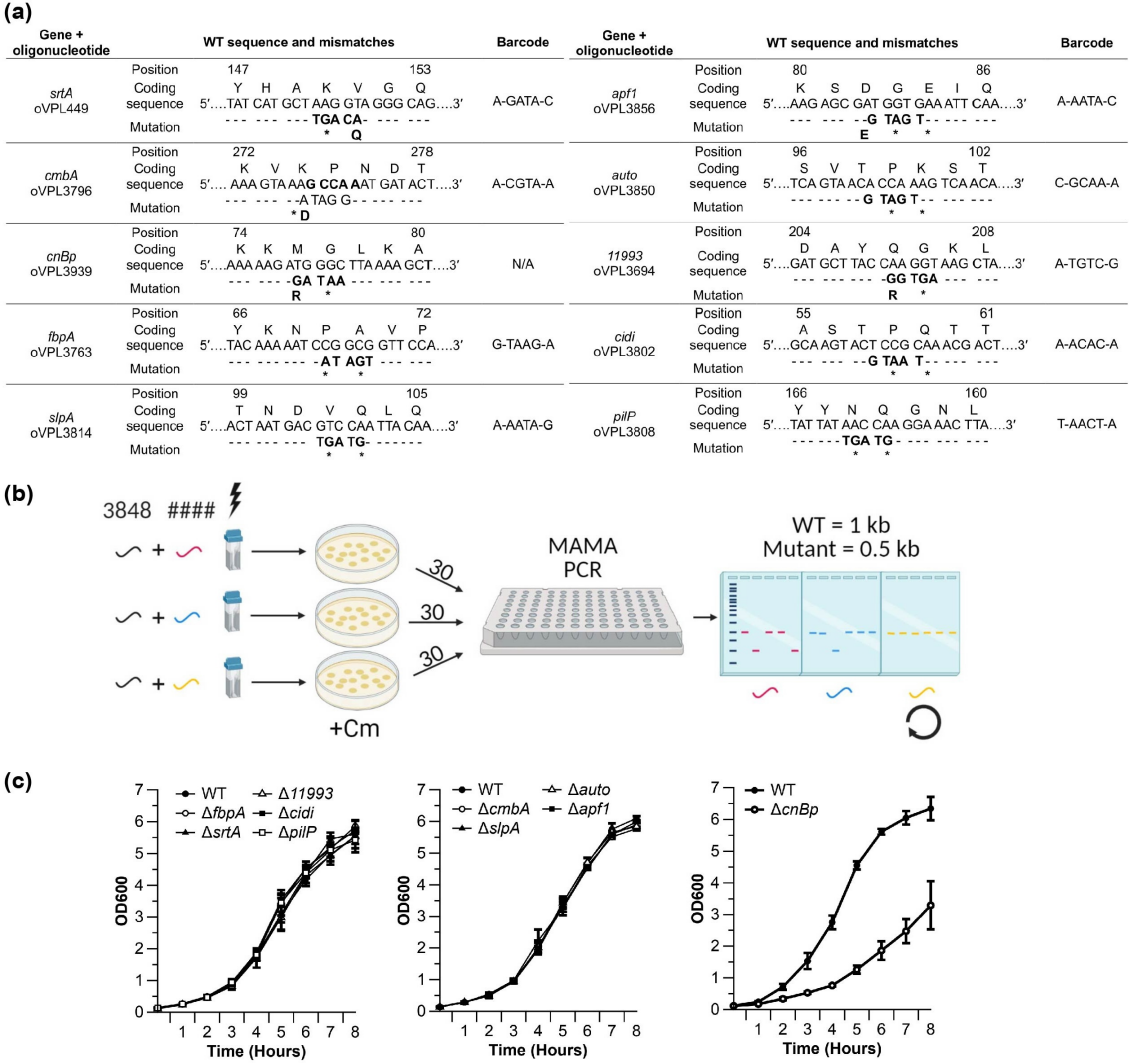
Construction, recovery, and growth analysis of adhesin mutants. (**a**) The gene targets and recombineering oligonucleotides used in this study are listed on the left. DNA sequences of the targeted adhesion proteins of *L. reuteri* are shown aligned with each recombineering oligonucleotide, with the encoded amino acid listed above. Numbers above the first and last amino acids encoded by each sequence indicate the amino acid positions. The directions of the lagging strands are indicated, and underneath are the mismatches in the recombineering oligonucleotides, which result in internal stop codons in each gene. Resulting amino acid mutations are indicated underneath the mismatched nucleotides. The coding strand is indicated in the third column, and barcodes derived from oVPL3848 are listed in the fourth column. (**b**) Scheme to optimize adhesin mutant recovery. Barcoding oligonucleotide oVPL3848 (3848, black) was dual-transformed into VPL4011 with an oligonucleotide targeting different adhesins (####; red, yellow, and blue) *via* electroporation. From the three transformations, colonies were selected on agar supplemented with chloramphenicol (Cm). Thirty CFUs from each transformation were screened for mutant genotypes by MAMA PCR. Wild-type genotypes have an expected size of 1 kb, while recombinant genotypes are 0.5 kb. If no recombinants were recovered from one of the transformations (yellow, in this example), the corresponding recombineering oligonucleotide was re-transformed alongside oligonucleotides targeting two additional adhesins. This process was repeated until each adhesin mutant was recovered. (**c**) Growth curves of all single mutants. Wild-type control (WT) is VPL4052, which contains the cat* gene insertion restored to *cat* with oVPL283. Data for WT are the same across the three growth curves, while the mutants are split across the three. The results shown are averages from three independent experiments ± standard error of the mean. right.

We applied the dual-recombineering concept to generate nine adhesion protein mutants, each with a unique barcode (Fig. 3a; Table 4). To optimize the mutant screening process, we developed a scheme to efficiently identify adhesin mutants (Fig. 3b). With the dual-recombineering method—in which we transformed strain VPL4011 with oVPL3848 and a recombineering oligonucleotide targeting a putative adhesion mutant—we typically recovered at least one recombinant per 30 CFUs screened (Fig. S1). If we did not recover a recombinant genotype after screening 30 CFU, the same experiment was repeated in the next round of transformations until the desired genotype was recovered (Fig. 3b). Nine adhesin mutants were recovered using this method, all with unique barcodes that restored cat*. One mutant, Δ*cnBp*, could not be obtained with this method, and was instead obtained with CRISPR-Cas9-assisted recombineering (22, 23).

After we confirmed all mutants and their barcodes by Sanger sequencing, we determined the growth rates for all strains. Growth rates were calculated from the resulting growth curves, and no differences were observed between nine mutants and the chloramphenicol-resistant wild-type control (VPL4052; Fig. 3c). However, Δ*cnBp* exhibited a growth defect and was thus excluded from further analysis (Fig. 3c). At this point, we were in a position to determine intestinal survival.

### Inactivation of genes encoding surface adhesins does not impact gastrointestinal survival

**Fig 4:**
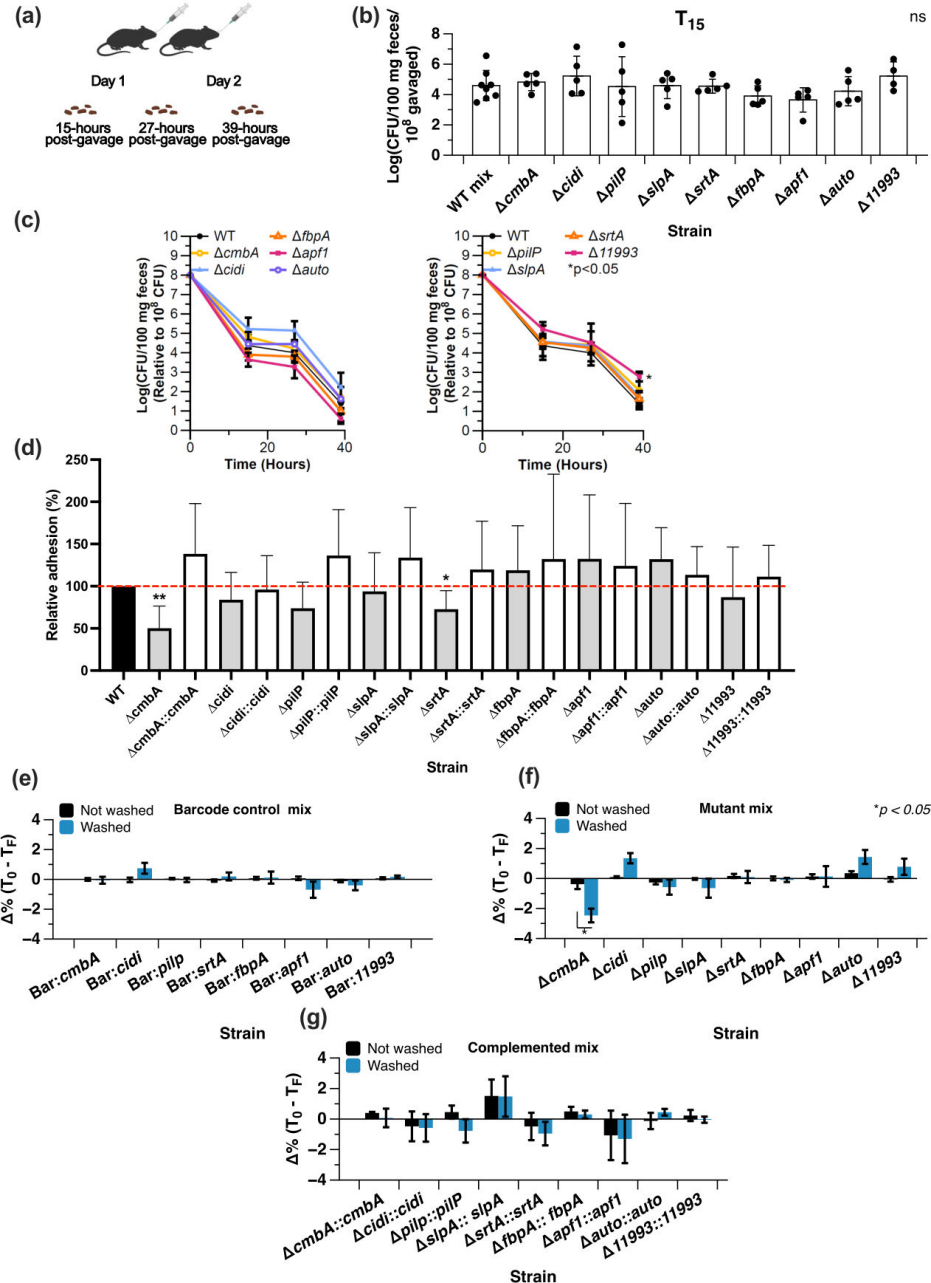
Mouse gastrointestinal survival of adhesin mutants and adhesion to human colon cancer cells. (**a**) Mice (*n* = 5–8) were administered with 10^8^ CFU of each mutant for two consecutive days. At 15, 27, and 39 h after the second gavage, fecal material was collected, resuspended to 100 mg/mL in PBS, and plated for quantification. (**b**) At 15 h, we measured the survival of each adhesion mutant following transit through the murine GI tract (*n* = 5). A mix of VPL4011 (*n* = 8) transformed with an oligonucleotide conferring each mutant barcode served as a control (WT mix). (**C**) Persistence of each mutant is depicted as the CFU recovered over the course of the *in vivo* experiment. Data for WT are the same across both graphs. (**d**) Relative percent adhesion of each mutant and its complemented strain to HT-29 cells was compared with chloramphenicol-resistant wild-type control (VPL4052) and empty vector control of *L. reuteri* (VPL31134), respectively. VPL1014 served as the WT control. The results shown are averages from six independent experiments with three technical replicates each, ±standard error of the mean, *; *P* < 0.05, **; *P* < 0.01, no statistical label; *P* > 0.05 (two-tailed unpaired *t*-test between adhesion mutant and complemented strain) (**e-g**) Change in relative ratio of each strain within each sample recovered from enteroid monolayers (T_F_) compared with the respective ratio of each strain in the starting mixture (**T_0_**). Data are presented as the change in relative percent (Δ%(T_F_- T_0_) for the barcode control mix (**e**), mutant mix (**f**), and the complemented mix (**g**) based on sequencing reads targeting the *cat* barcode. Positive numbers indicate an increase in relative ratio while negative numbers indicate a decrease. The results shown are averages from three independent experiments with three technical replicates each, ±standard error of the mean; *, *P* < 0.05.

To determine gastrointestinal survival, we administered mice (*n* = 5–8 mice/group) by oral gavage with each mutant and VPL4052 for two consecutive days with 10^8^ CFU per day. Fifteen hours after the second gavage, we recovered *L. reuteri* and its mutants from the fecal material, which was repeated at 27 and 39 h (Fig. 4a). Average recovery at 15 h ranged from 10^4^ CFU/100 mg feces (Δ*fbpA* and Δ*apf1*) to 10^5^ CFU/100 mg feces (Δ*cmbA*, Δ*cidi* and Δ*11993*) (Fig. 4b). Compared with the control (VPL4052; 10^5^ CFU/100 mg feces), survival of the mutants was not significantly different (*P* > 0.5).

We also tested the ability of each adhesin mutant to thrive in the gut by tracking persistence. Although we observed variation in the level by which mutants were recovered over time (Fig. 4c), no significant differences were observed up until the 27 h time point. At the final time point (39 h), 2–3 log fewer CFUs were recovered compared with t = 27 h, with exception of Δ*11993*, which decreased by just under 2–log (1.73 ± 0.53; *P* < 0.05). Thus, with exception of ∆*11993*, intestinal survival and persistence of individual mutants were not signifantly reduced.

### Inactivation of *cmbA* and *srtA* reduced adhesion to human colon cancer cells

As a first step toward understanding the extent to which each of the mutant strains adheres to epithelial cells, we performed an *in vitro* adhesion assay with the colonic cell line HT-29. All nine single mutants, their complemented derivatives and VPL4052 (chloramphenicol-resistant control) were added to HT-29 monolayers at a 5:1 bacteria:epithelial cell ratio, and the relative percent adhesion was calculated as described previously (46). Strains Δ*cmbA* (an SDP) and Δ*srtA* (sortase) adhered significantly less to HT-29 compared with VPL4052, while the remaining seven mutants adhered to HT-29 at levels comparable to VPL4052 (*P* > 0.05; Fig. 4d). For the seven mutants which adhered similar to VPL4052, the question arises if these genes are expressed under these experimental conditions. To determine gene expression, we performed RNAseq analysis. *L. reuteri* VPL1014 was cultured and harvested in an identical manner as when cells were prepared for the HT-29 cell adhesion assays. We determined that *pilP* and *11993* have low relative expression (>10 < 20 counts per million mapped [CPM] reads) (Fig. S2), while the other seven genes are expressed at higher levels (>91 < 5547; Fig. S2). Of all nine target genes, transcript levels of *cmb* were the second highest (~1,200 CPM reads), whereas expression levels of *slpA*, *srtA*, *fbpA*, *apf1*, and autolysin were comparable (~250 CPM reads). Interestingly, although not involved in adhesion to HT-29, highest relative expression was observed for *cidi* (>5,000 CPM reads). Thus, the roles of PipP and 11993 in adhesion to HT-29 are unknown under these experimental conditions. That CmbA and SrtA are important for adhesion to HT-29 was further validated by our complemented strains, which restored the adhesive ability of the respective mutants. That several knockouts did not reduce adhesion to HT-29 cell does not exclude the possibility that (some of the) encoded proteins play a role in adhesion in the host, especially given the fact that HT-29 cells produce little to no mucus (65).

Human intestinal organoid-derived epithelial derived monolayers (HIODEM model) contain mucus-producing goblet cells in addition to enterocytes and antigen sampling M cells (66–69). For adhesion experiments, we designed a competition experiment mixed them in which bacterial strains were mixed at a 1:1:1:….:1 ratio. We used mixtures of complemented mutants and barcoded VPL4011 as controls. All the mixtures adhered similarly (*P* > 0.3) to the monolayers, specifically 1.14 ± 0.66% barcode control vs 2.01 ± 1.3% mutant mix vs. 1.34 ± 0.13% complemented mix (Fig. S3).

To gain insight into the adhesive ability of each individual strain within the mixture, we determined the relative abundance of each mutant. First, the samples were lysed by bead beating, and the total DNA was harvested and prepared for next-generation sequencing (NGS) targeting the *cat* gene barcodes. We compared the percentage of reads corresponding to each barcode in the mixtures just prior to adding them to the enteroid monolayers (T_0_) to the percentage of barcode reads recovered from the adhesion assay (T_F_) (Fig. 4e). A positive change in percentage indicates that the relative proportion of a mutant increased in the population, while a negative change in percentage indicates a decrease in relative proportion. Mixtures added to unwashed cells served as controls to compare percent changes between T_F_ and T_0_ of the washed cells. As expected, the ratio of strains in the barcode control mix (Fig. 4e) did not change during the experiment (percent changes were all between ± 0.70%), indicating that each strain within the control mixture was equally capable of adhering to the monolayers. Importantly, the ratio of strains in the unwashed control also did not change (all percent changes between ± 0.1%), which serves as an indication for the sensitivity and accuracy of the sequencing method (Fig. 4e and f). For the mutant mixture, only Δ*cmbA* exhibited a significant decrease in ratio compared with the Δ*cmbA* unwashed control (−2.46 ± 0.46% vs 0.46 ± 0.33%, respectively, *P* < 0.05) (Fig. 4f). Two mutants, Δ*cidi* (1.34 ± 0.34%) and Δ*auto* (1.43 ± 0.46%) increased (though not significantly) in relative ratio following the adhesion assay (Fig. 4f). Unexpectedly, Δ*srtA* did not exhibit a decrease in relative proportion between T_F_ and T_0_ (0.10 ± 0.4%). The complemented mixture exhibited higher variance than the barcode and mutant mixes (Fig. 4g). Despite this, the ratios of each strain between the washed and not washed samples are very similar, and with the exceptions of Δ*apf1*, Δ*slpA* and Δ*srtA*, most T_F_ ratios remained very similar to the respective T_0_ ratios (Fig. 4g). Altogether, these data show that CmbA is a major adhesin in these experimental designs.

### A nonuple mutant has reduced capacity to adhere to enteroid monolayers

Our data show that in the adhesion models we have applied, most gene knockouts putatively encoding adhesins do not significantly reduce the adhesive ability of *L. reuteri*. Several homologs in other bacteria did reveal a role in microbial adhesin. Also, as stated before, it cannot be excluded that there is a role for bacterium–host interactions in the human GI tract (50–52, 55, 57, 58, 60). With this in mind, we decided to include all nine genes putatively encoding adhesins to reduce the potential of long-term colonization.

**Fig 5:**
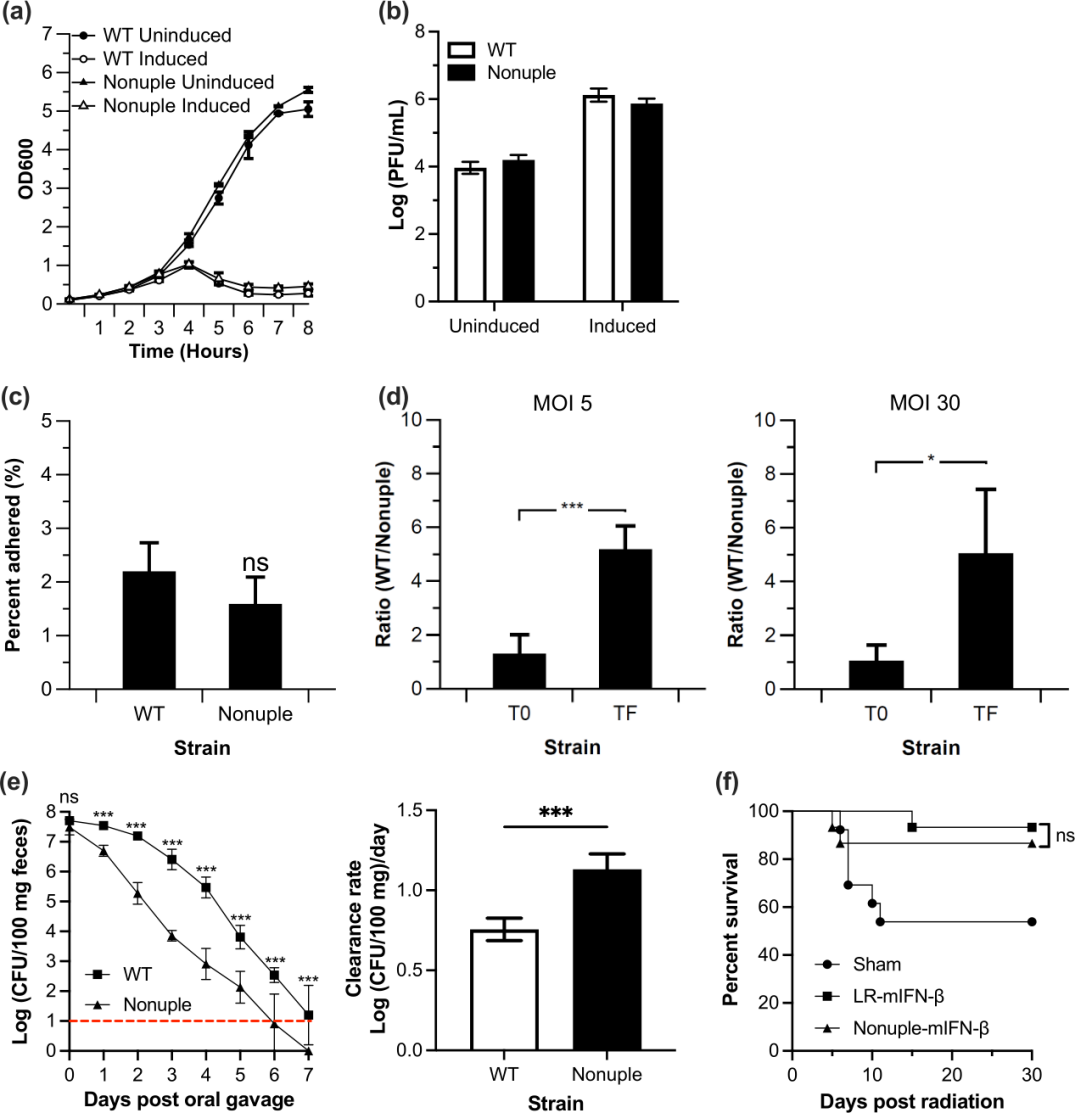
*In vivo* characterization and therapeutic efficacy of the nonuple mutant. (**a**) Growth curve and mitomycin C induction of *L. reuteri* VPL1014 (WT) and nonuple mutant. (**b**) At the endpoint of the growth experiment (**T_8_**), samples derived from uninduced and induced cultures of *L. reuteri* VPL1014 (WT) and the nonuple variant were processed to quantify phage production (PFU) (*P* > 0.3). (**c**) Adhesion of *L. reuteri* VPL1014 (WT) and nonuple mutant to monolayers of HT-29 cells (*P* > 0.4). (**d**) Adhesion competition experiment on human enteroid monolayers between wild-type control (WT) and the nonuple mutant. Multiplicities of infection (MOI) ratios of 5:1 and 30:1 were tested. Results are expressed as a ratio of wild-type CFU recovered compared with nonuple CFU recovered from the adhesion assay. T0 and TF represent the ratio of WT and nonuple cells before and after the adhesion assay, respectively. (**a-b**) The results shown are averages from three independent experiments ± standard error of the mean. For panels (**c**) and (**d**), the results shown are averages from three independent experiments (with three technical replicates each) ±standard error of the mean. *, *P* < 0.05; ***, *P* < 0.005. (**e**) Persistence of WT and the nonuple mutant in the mouse GI tract. Male B6 mice (*n* = 6/group) were orally gavaged with 100 µL (10^9^ CFU/mouse) WT or nonuple mutant, and persistence of each bacteria was monitored daily for7 days post-oral gavage. The clearance rate was determined from absolute slope value of linear curve between bacterial concentration [Log (CFU/100 mg)] and post-oral gavage days, constructed using data from 0 to 5 days post-oral gavage. ***, *P* < 0.001 (two-tailed unpaired *t*-test). (**f**) Survival of mice exposed to partial body irradiation (PBI) was comparable between the treatment groups LR-IFN-β and nonuple-IFN-β. Twenty-four hours following PBI (13.35 Gy), female C57BL/6 mice (*n* = 15/group) were gavaged with 200 µL saline (sham), LR-IFN-β, or nonuple-IFN-β, each 10^9^ CFU. The Survival of animals was monitored for 30 days. Significance in survival was determined by a two-sided log-rank test with *P* < 0.05 regarded as statistically significant.

A nonuple mutant without the chloramphenicol resistance gene expression cassette was obtained by sequentially transforming each adhesion mutant’s recombineering oligonucleotide into a single strain. A double mutant, triple mutant, quadruple mutant, and so-on were obtained until all nine mutations were in a single strain, resulting in the nonuple mutant (VPL4366). Because our ultimate goal is to employ the nonuple strain as a delivery vehicle platform, we determined growth kinetics and phage production to test that the combined mutations did not confer a growth defect or impact the delivery mechanism of phage-mediated lysis (33). Growth of the nonuple mutant was similar compared with the wild type, with doubling times of 1.15 ± 0.03 and 0.99 ± 0.05 doublings/h, respectively (*P* > 0.05; Fig. 5a). Phage levels of wild type and the nonuple derivative were similar [(3.96 ± 0.18 log(PFU/mL) vs 4.20 ± 0.14 log(PFU/mL), respectively; *P* > 0.3)] (Fig. 5b). Also, basal level phage production was similar [(6.12 ± 0.19 log(PFU/mL) and 5.86 ± 0.15 log(PFU/mL) for the wild type and recombinant strain, respectively; (*P* > 0.3)]. Thus, the growth and phage production profiles of the nonuple mutant were similar to that of wild type.

Next, we tested the ability of the nonuple derivative to adhere to HT-29 cells. Strains were co-incubated with a confluent monolayer of HT-29 (MOI 5:1). Percent adhesion of the nonuple mutant (1.59 ± 0.5%) was comparable to that of the wild type (2.2 ± 0.3%; *P* > 0.4) (Fig. 5c). However, using the enteroid assay model, the control strain significantly outcompeted the nonuple derivative (fivefold; Fig. 5d), which is thus indicative of reduced adhesive ability when using a MOI of 5:1 or 30:1. At this point, we were able to compare the therapeutic efficacy between *L. reuteri* wild type and the nonuple variant.

### The nonuple mutant was cleared faster from the mouse gastrointestinal (GI) tract than the wild type

To assess the persistence of nonuple mutant compared with wild type, we administered by oral gavage the wild-type or the nonuple mutant to conventional mouse and quantified each strain from feces each day for 7 days. Compared with the wild type, the nonuple mutant was cleared from the mouse GI tract faster (*P* < 0.001) and could not be detected anymore in the feces 6 days post-oral gavage (Fig. 5e).

### Therapeutic efficacy is comparable between the nonuple mutant and the wild-type background

To test if the therapeutic efficacy of the nonuple variant is comparable to the wild-type background, we used a previously established model in which engineered *L. reuteri* releasing IFN-β promoted survival of mice exposed to irradiation (37). Compared with the sham group, survival was significantly higher in mice that received WT-IFN-β (*P* < 0.05), whereas survival was comparable between mice treated with WT-IFN-β or nonuple-IFN- β (*P* = 0.4; Fig. 5e). Thus, compared with the wild type, the nonuple mutant is expected to have reduced colonization potential, yet the therapeutic efficacy is comparable in a model of irradiation mitigation.

## DISCUSSION

One of our goals is to develop a therapeutic probiotic bacterium that has reduced ability to colonize the human intestinal tract. We constructed 10 adhesin mutants in *L. reuteri* VPL1014, which we tracked by a unique barcode in the chromosome. Nine of 10 selected adhesin mutants (Δ*srtA*, Δ*slpA*, Δ*cmbA*, Δ*auto*, Δ*apf1*, Δ*pilP,* Δ*11993*, Δ*fbpA*, and Δ*cidi*) demonstrated no negative effect on growth or *in vivo* survival, and two mutants (Δ*srtA* and Δ*cmbA*) adhered significantly less to intestinal epithelial cells. We developed a nonuple mutant, which was significantly deficient in its ability to adhere to human colonoid monolayers, and was cleared faster from the mouse GI tract compared with the wild type. This nonuple strain will serve as an important first step towards accomplishing biological containment in *L. reuteri*.

To enable the direct comparison of the adhesion mutants in *in vitro* and *in vivo* settings, we devised a barcoding scheme that generates unique tags in the *cat* gene of each mutant. Our design can theoretically generate a total of 3,904 unique barcodes yielding a functional *cat* gene. However, in the initial screen, we observed that 81 of 96 recombinant genotypes were unique, which suggests bias towards either bases at the wobble positions or codons that replace the stop codon. One possibility is that recombination with the non-optimized oVPL3848 biased the results by failing to evade the DNA mismatch repair system (MMR) at the wobble base positions. At wobble base positions, the degenerate oligonucleotide introduces a single mismatch that is most likely detected by MMR, as only five adjacent mismatches have demonstrated full avoidance of MMR in *L. reuteri* (22). It is also plausible that certain amino acids may not restore gene function, as we observed codons that replaced the stop codon translate to only 8/20 amino acids. If the limited number of amino acids at the stop codon position relates to functionality or folding rather than recombination efficiency of oVPL3848, the total potential barcodes would decrease to 1,536. Despite the potential limitation of only a few amino acids restoring Cat*,* 85% of the 96 oVPL3848 transformants sequenced contained unique barcodes. Additionally, the barcode is within a gene conferring antibiotic resistance, and it is also possible that random mutations may arise in the barcode during growth, resulting in background sequencing reads in downstream experiments. However, we observed that less than 5% of total reads in the competition experiment were non-mutant barcode sequences, indicating that—at least in *L. reuteri* VPL1014—this is not a concern. Therefore, the dual-recombineering method is suitable to generate barcoded libraries in a quick and efficient manner in *L. reuteri*.

Following mutant construction, we observed that Δ*cmbA* and Δ*srtA* exhibited lower adhesion to HT-29 cells compared with wild type, which is in-line with the results from Jensen *et al.,* while the decrease in nonuple mutant adhesion to HT-29 was not statistically significant compared with that of wild type. Single mutants, Δ*auto*, Δ*fbpA* and Δ*apf1,* each resulted in a modest (non-significant) increase in adhesion. It is therefore not unreasonable to restore each of these genes in the nonuple to assess if the adhesion to HT-29 is impacted. At the same time, although HT-29 cells are well characterized and broadly used as a model system for studying adhesion and host-microbe interactions (70, 71), it remains an *in vitro* system that has limitations, including a lack of diversity in cell type and structure, and limited mucus production. An alternative model to study probiotic–host interactions is based on HIODEM cells. Monolayers derived from enteroids are composed of a variety of cell types, including mucus-producing goblet cells, Paneth cells, and hormone-secreting enteroendocrine cells, which create a more *in vivo-*like environment than cell lines (72). Compared with traditional cell lines, like HT-29, culturing enteroid monolayers is more costly and laborious (73). Therefore, rather than testing each mutant one by one, we instead devised a competition experiment in which we assayed a mixture of all mutants for adherence in a manner similar to previous adhesin studies (74, 75). A pooled adhesion competition assay allows for direct comparison of the adhesion ability of each mutant relative to each other. Inactivation of *cmbA* resulted in a significant reduction in adhesion compared with the other mutants, including sortase. This was an unexpected finding, and it cannot be excluded that strain–strain interactions within the mixture could have contributed to this result. A side-by-side comparison between adhesion assays based on strain mixtures and individual strains is needed to further elucidate this. Importantly, variation across the replicates was generally very low in this experiment, validating the barcoding method to distinguish these strains with a high level of sensitivity in a pooled competition assay.

We observed that the nonuple mutant has reduced capacity to adhere to enteroid monolayers when in direct competition with the wild-type strain. Whereas we thus-far identified CmbA as the major adhesin in *L. reuteri, the* level by which the wild-type strain outcompetes the nonuple strain suggests there may be a cumulative effect with regards to the contribution other adhesins make.

One limitation of this study is that we did not investigate the immunogenic properties of the nonuple mutant compared with wild type. Future studies should explore to what extent these adhesins affect the potential of *L. reuteri* VPL1014 to stimulate the immune system or probiosis in general. The immunogenic properties of these adhesins could also impact the magnitude of efficacy of certain effector molecules because some adhesins may act as adjuvants (76, 77). For some applications of probiotics or effector molecule delivery by bacteria, it may be ideal for the delivery vehicle to lack immunomodulatory effects that may be detrimental in certain disease states, such as in patients with severely compromised immune systems (78, 79). On the other hand, adhesin-mediated interactions with the epithelium or mucus—such as CmbA-mediated adhesion by *L. reuteri—may*—may be important to deliver proteins and peptides systemically (31, 32). This means that, depending on the application, the development of recombinant microbes may have to be tailored to maximize therapeutic efficacy. In a model of mitigation of radiation-induced injury both WT-IFN-β and nonuple-IFN-β offered similar protection, suggesting that at least in this preclinical model the adhesins are not important for therapeutic efficacy.

In future studies, we will examine the persistence and colonization abilities of the nonuple mutant in *in vivo* models such as germ-free mice and non-human primates. We predict that these *in vivo* models will provide further insight into the utility of the nonuple mutant as a therapeutic delivery platform. Overall, our work represents another step toward the development of *L. reuteri* as a therapeutic delivery vehicle for clinical use.

## MATERIALS AND METHODS

### Bacterial strains and growth conditions

The bacterial strains and plasmids used in this study are listed in Table 2. *Escherichia coli* EC1000 and *Lactococcus lactis* MG1363 were used as intermediate cloning hosts. *E. coli* was cultured aerobically at 37°C in lysogeny broth (LB; Teknova), and *L. lactis* was cultured statically at 30°C in M17-broth (Difco; BD BioSciences) supplemented with 0.5% (w/v) glucose. Competent cells of *E. coli* EC1000 and *L. lactis* MG1363 were prepared as described previously (80, 81). *L. reuteri* was grown in De Man, Rogosa, and Sharpe (MRS) medium (Difco, BD Biosciences) under hypoxic conditions (5% CO_2_, 2% O_2_) at 37°C. *L. reuteri* competent cells were prepared as described previously (23). As needed, erythromycin was supplemented at 5 µg/mL for the *L. reuteri* strains and 300 µg/mL for *E. coli* EC1000. Chloramphenicol was added as needed at 5 µg/mL for *L. reuteri* and *L. lactis*. Tetracycline was added as needed at 25 µg/mL for *L. reuteri* and 10 µg/mL for *L. lactis*.

**TABLE 2:** Bacterial strains and plasmids used in this study.

Strains (name/VPL)^*a*^	Characteristics^*b*^	Source/reference^*c*^
*E. coli* EC1000	Derivative of *E. coli* MC1000 in which *repA* is integrated in chromosome	(82)
*L. lactis* MG1363	Plasmid-free derivative of *L. lactis* subsp. *cremoris* NCDO712	(83)
*L. reuteri* VPL1014	Derivative of L. *reuteri* ATCC PTA 6475 wild type	Laboratory stock
VPL3187	Mutant harboring pVPL3004 and pVPL3016	(23)
VPL4011	Mutant with inactivated *cat* (cat*, L141*) gene insertion	This work
VPL4018	Δ*srtA*::oVPL449 (K150*V151Q)	(23)
VPL4052	Mutant with cat* restored to functional *cat* with oVPL428	This work
VPL4359	Derivative of VPL4011; Δ*cmbA*::oVPL3796 (P282*N283D); *cat*::oVPL3848	This work
VPL4360	Derivative of VPL4011; Δcyclic-phosphodiesterase (Δcidi)::oVPL3802 (P58*Q59*); *cat*::oVPL3848	This work
VPL4361	Derivative of VPL4011; Δ*pilP*::oVPL3808 (N162*Q163*); *cat*::oVPL3848	This work
VPL4362	Derivative of VPL4011; Δ*slpA*::oVPL3814 (V102*Q103*); *cat*::oVPL3848	This work
VPL4363	Derivative of VPL4011; Δ*srtA*::oVPL449 (K150*V151Q); *cat*::oVPL3848	This work
VPL4364	Derivative of VPL4011; Δ*fbpA*::oVPL3763 (N68*P69*); *cat*::oVPL3848	This work
VPL4365	Derivative of VPL4011; Δ*apf1*::oVPL3850 (D82EG83*E84*); *cat*::oVPL3848	This work
VPL4367	Derivative of VPL4011; Δautolysin (Δauto)::oVPL3856 (P99*K100*); *cat*::oVPL3848	This work
VPL4368	Derivative of VPL4011; ΔLAR_0044 (Δ*11993*)::oVPL3694 (Q211RG212*); *cat*::oVPL3848	This work
VPL4366	Mutant with nine inactivated putative adhesion genes; nonuple	This work
VPL4379	Derivative of *L. reuteri* VPL1014; Δ*cnBp*::oVPL3939 (M76RG77*)	This work
Plasmids		
pJP042	ssDNA recombineering plasmid, Em^R^ derivative of pSIP411 in which the *gusA* gene is replaced with *recT1* derived from *L. reuteri* ATCC PTA 6475. *recT1* is under the control of an inducible promoter	(22)
pVPL3583	pJP028 vector	(33)
pVPL3002	pORI19 harboring *L. reuteri* derived *ddl*F258Y^*d*^	(24)
pVPL3004	Em^R^, derivative of pNZ9530 in which *nisR* and *nisK* genes were replaced with the tracrRNA, *cas9*and CRISPR array derived from pCAS9	(23)
pVPL3017	ssDNA recombineering plasmid, Cm^R^ derivative of pJP042	Laboratory stock
pVPL3115	Derivative of pNZ8048 harboring the CRISPR array	(23)
pVPL3031	Derivative of pNZ8048 harboring cat* and *ery*.	This work
pVPL3038	pVPL3002 derivative. Suicide shuttle vector with flanking sequence of a non-coding region in *L. reuteri* designed for chromosomal insertions	(31)
pVPL3047	pVPL3038 derivative with inactivated cat* integration cassette	This work
pVPL31134	pJP028 derivative, pCtl-ThyA	(33)
pVPL31464	pJP028 derivative, pIL-22-ThyA	(24)
pVPL31467	pJP028 derivative; *cmbA* complementation plasmid	This work
pVPL31514	pJP028 derivative, *pilP* complementation plasmid lacking *cat* gene	This work
pVPL31515	pJP028 derivative, *slpA* complementation plasmid lacking *cat* gene	This work
pVPL31516	pJP028 derivative, *srtA* complementation plasmid lacking *cat* gene	This work
pVPL31517	pJP028 derivative, *fpbA* complementation plasmid lacking *cat* gene	This work
pVPL31518	pJP028 derivative, autolysin (auto) complementation plasmid lacking *cat* gene	This work
pVPL31519	pJP028 derivative, *apf1* complementation plasmid lacking *cat* gene	This work
pVPL31520	pJP028 derivative, *11993* complementation plasmid lacking *cat* gene	This work
pVPL31521	pVPL31467 derivative lacking *cat* gene	This work

^*a*^ VPL: van Pijkeren Lab strain identification number. pVPL: Van Pijkeren Lab plasmid identification number.

^*b*^ repA: replication initiation protein; Em^R^: erythromycin-resistant; Cm^R^: chloramphenicol-resistant.

^*c*^ Nonsense mutation; *cat*: chloramphenicol acetyltransferase; *ery*: 23S ribosomal RNA methyltransferase; LAR_#### refer to closed reference genome *Limosilactobacillus reuteri* JCM1112.

^*d*^ *ddlA*: d-alanine-d-alanine ligase (LAR_1277).

### Cell line and organoid culture

The human colorectal adenocarcinoma cell line HT-29 (ATCC HTB-38) was obtained from the American Type Culture Collection. HT-29 cells were grown in Dulbecco’s modified Eagle’s medium containing 4.5 g/L glucose with L-glutamine and sodium pyruvate (VWR, 45000–304) supplemented with 10% (v/v) heat-inactivated fetal bovine serum (Sigma-Aldrich, 12,306 C-500ML). The cells were maintained at 37°C in a humidified atmosphere of 5% CO_2_ and sub-cultured when the cells reached 70–90% confluence through visual observation under microscope.

Human colon cancer enteroids, a type of organoids derived from the small intestine, (121 CRC; it is a limited stage colon cancer stage IIC which was resected. Molecular profiling at DNA level by Qiagen cancer panel testing revealed: APC E1309fs; KRAS G12D; TP53 R175H; HNF1A T156M) were isolated and cultured as described previously (84). Briefly, human tissue from needle biopsy or surgical resection was placed in chelation buffer and then digested in stock media: advanced DMEM/F12 medium (Invitrogen, Carlsbad, CA) containing 10% fetal bovine serum, collagenase (1 mg/mL), dispase (12.5 µg/mL) and 1% (v/v) penicillin and streptomycin. The tissues were disrupted with intermittent shaking. The cell suspension was then separated from digestion buffer through centrifugation at 300×*g* at 4 °C for 5 min and then washed once with 1×PBS. PBS was removed from the cell pellet and the pellet was resuspended in DMEM/F12 containing 1× glutamax, 10 mM HEPES, and 1% (v/v) penicillin and streptomycin. Cell suspensions were maintained on ice and mixed with Matrigel at a 1:1 ratio before being plated as droplets onto 24-well culture plates and incubated at 37°C. Plates were inverted after 2 to 3 min of incubation. After the mixture had solidified, cultures were overlaid with feeding medium consisting of 50% (v/v) stock media and 50% (v/v) conditioned medium obtained from WNT3a L cell line (ATCC CRL-2647) mixed with EGF (50 ng/mL). To maintain enteroids, media were changed every other day.

### Bioinformatic analyses

Building on the analyses conducted by Saulnier *et al*. and Jensen *et al*., we included previously identified SDPs encoding genes in our study (49, 85). Genes previously identified as pseudogenes were excluded from further analysis (49). Homologs of the proteins that were well known to play a role in adhesion, such as those described by Jensen *et al*. were used as a query to search the *L. reuteri* JCM1112 chromosome using BLASTp at the National Center for Biotechnology Information website (http://www.ncbi.nlm.nih.gov) (see Table 1 for accession numbers used as queries and references). Adhesin homologs were also analyzed for SDP characteristics. The sorting motif LPxTG was manually searched for in the protein sequences. InterPro, a database of protein families, was used to search for YSIRK-G/S signal sequences (pfam04650), cell wall anchor domains (TIGR01167) and other protein domains (86). Secretion signal peptides were predicted with SignalP 5.0 (64), and transmembrane helices were predicted with TMHMM 2.0 (http://www.cbs.dtu.dk/services/TMHMM). Repeats in the protein sequences were identified using RADAR (http://www.ebi.ac.uk/Tools/pfa/radar).

### Generation of in-frame stop codon in chloramphenicol acetyltransferase

Plasmid pJP028 harbors *cat* and *ery*, which encode chloramphenicol and erythromycin resistance, respectively. Expression of *cat* is placed under the control of the P_HELP_-promoter (87). Oligonucleotides oVPL261-262 (Table 3) are complementary to the *cat* gene with exception of three bases; subsequent amplification and self-ligation of the amplicon yielded pVPL3031 which contains an in-frame stop codon in *cat* (cat*; L141*).

**TABLE 3:** Oligonucleotides used for plasmid construction in this study

Oligonucleotides^*a*^	Sequence (5’−3’)	Target/comment^*b*^
VPL202	atgaactttaataaaattgatttagac	Fwd, *cat* gene from pVPL3031
VPL203	ttataaaagccagtcattaggcc	Rev, *cat* gene from pVPL3031
VPL261	TCAagaaaaagcattttcaggtatagg	Fwd, introduces internal stop codon into *cat* from pVPL3031, generating cat*(L141*)
VPL262	tctattattccttggacttcattt	Rev, introduces internal stop codon into *cat* from pVPL3031, generating cat*(L141*)
VPL271	ttaaaaattaatctttccagtaataatcaacatc	Fwd, internal oligo for pVPL3038 backbone for cloning cat* gene
VPL272	ttaaaatgtaggtttaatttttagggc	Rev, internal oligo for pVPL3038 backbone for cloning cat* gene
VPL283	aagcagtcaaaaagccctaaaaattaaacctacattttaacattatgctttggcagtttattcttgacatg	Fwd, olig with 40 bp clamps to amplicon 265–266 to clone cat* gene (lagging strand orientation)
VPL284	tgcgctgatgttgattattactggaaagattaatttttaatttgattgatagccaaaaagcagcag	Rev, olig with 40 bp clamps to amplicon 265–266 to clone cat* gene (lagging strand orientation)
VPL309	tctcgctttgattgttctatcgaaag	Rev, for amplifying pJP028 backbone omitting *cat* gene
VPL310	ataaggaagataaatcccataagggc	Fwd, for amplifying pJP028 backbone omitting *cat* gene
VPL334	aactttcgccattaatgtgttttatcgg	Fwd, for single-crossover and double-crossover screening of cat* insertion
VPL335	agacagatgacaagccctttagc	Rev, for single-crossover and double-crossover screening of cat* insertion
VPL363	taatatgagataatgccgactgtac	Fwd, for screening for presence of pThyA-Ctl and pIL-22-ThyA plasmids
VPL728	ttcattacatccatgggtgtc	Rev, for screening for presence of pThyA-Ctl and pIL-22-ThyA plasmids
VPL736	tgaatgagtgagtcaacttg	Fwd, for amplifying pMutL promoter sequence from pSIP411:pMutL-ThyA
VPL737	taaatatcaccttatttcaa	Rev, for amplifying pMutL promoter sequence from pSIP411:pMutL-ThyA
VPL1286	tgatctttgaaccaaaattag	Fwd, for amplifying pJP028 backbone
VPL1408	agaaaaccgactgtaaaaagtacag	Rev, for amplifying pJP028 backbone
VPL4033	atgctatcaagaaaaaattataagga	Fwd, for amplifying *cmbA* (LAR_0958) gene
VPL4034	ctaatcatgtttacgcttcttgcc	Rev, for amplifying *cmbA* (LAR_0958) gene
VPL4035	gcagcagaaattgaaataaggtgatatttaatgctatcaagaaaaaattataaggaaact	LCR bridging oligo for ligating pMutL promoter sequence to *cmbA* for insertion into pJP028 for complementation
VPL4036	gccgactgtactttttacagtcggttttcttgaatgagtgagtcaacttgaattatttgc	LCR bridging oligo for insertion of pMutL into pJP028 for adhesion protein complementation
VPL4037	ggtttgggcaagaagcgtaaacatgattagtgatctttgaaccaaaattagaaaaccaag	LCR bridging oligo for insertion of *cmbA* into pJP028 for complementation
VPL4038	gtgaaaaaagataaaaagcga	Fwd, for amplifying *srtA* (LAR_0227) gene
VPL4039	ttaacgacctgtcgtatatt	Rev, for amplifying *srtA* (LAR_0227) gene
VPL4040	gcagcagaaattgaaataaggtgatatttagtgaaaaaagataaaaagcgatcatttgaa	LCR bridging olig for ligating pMutL promoter sequence to *srtA* for insertion into pJP028 for complementation
VPL4041	tttagcgaaaaatatacgacaggtcgttaatgatctttgaaccaaaattagaaaaccaag	LCR bridging olig for insertion of *srtA* into pJP028 for complementation
VPL4045	atgtcttttgacggcttg	Fwd, for amplifying *fbpA* (LAR_0878) gene
VPL4046	ttagttagaaagtttatgcggtgt	Rev, for amplifying *fbpA* (LAR_0878) gene
VPL4047	tgcatgagtaaacaagccgtcaaaagacattaaatatcaccttatttcaatttctgctgc	LCR bridging oligo for ligating pMutL promoter sequence to *fbpA* for insertion into pJP028 for complementation
VPL4048	tatgtaacaccgcataaactttctaactaatgatctttgaaccaaaattagaaaaccaag	LCR bridging oligo for insertion of *fbpA* into pJP028 for complementation
VPL4049	atgaagaataatagttcaaaatattg	Fwd, for amplifying cyclic-phosphodiesterase (*cidi*) (LAR_0983) gene
VPL4050	ttaagcatgtttacgctt	Rev, for amplifying cyclic-phosphodiesterase (*cidi*) (LAR_0983) gene
VPL4051	gcagcagaaattgaaataaggtgatatttaatgaagaataatagttcaaaatattgttta	LCR bridging oligo for ligating pMutL promoter sequence to *cidi* for insertion into pJP028 for complementation
VPL4052	attattgatcgcaagcgtaaacatgcttaatgatctttgaaccaaaattagaaaaccaag	LCR bridging oligo for insertion of cidi into pJP028 for complementation
VPL4053	atgaagaaaagaaaatta	Fwd, for amplifying *pilP* (LAR_0989) gene
VPL4054	ttattcgtaccgtttaa	Rev, for amplifying *pilP* (LAR_0989) gene
VPL4055	gcagcagaaattgaaataaggtgatatttaatgaagaaaagaaaattaaagaagagttta	LCR bridging olig for ligating pMutL promoter sequence to *pilP* for insertion into pJP028 for complementation
VPL4056	attggggcaacacttaaacggtacgaataatgatctttgaaccaaaattagaaaaccaag	LCR bridging oligo for insertion of *pilP* into pJP028 for complementation
VPL4057	atgtcgaagaacaatgcac	Fwd, for amplifying *slpA* (LAR_1193) gene
VPL4058	tcagtaatagttgggtttatctgt	Rev, for amplifying *slpA* (LAR_1193) gene
VPL4059	gcagcagaaattgaaataaggtgatatttaatgtcgaagaacaatgcacaagaatatgta	LCR bridging oligo for ligating pMutL promoter sequence to *slpA* for insertion into pJP028 for complementation
VPL4060	gggatgacagataaacccaactattactgatgatctttgaaccaaaattagaaaaccaag	LCR bridging oligo for insertion of *slpA* into pJP028 for complementation
VPL4061	gtgactaataaaaagcatta	Fwd, for amplifying autolysin, (auto) (LAR_1284) gene
VPL4062	ttagaattcaccataatat	Rev, for amplifying autolysin, (auto) (LAR_1284) gene
VPL4063	gcagcagaaattgaaataaggtgatatttagtgactaataaaaagcattataaattatat	LCR bridging oligo for ligating pMutL promoter sequence to autolysin for insertion into pJP028 for complementation
VPL4064	ttggtaagcctatattatggtgaattctaatgatctttgaaccaaaattagaaaaccaag	LCR bridging oligo for insertion of autolysin into pJP028 for complementation
VPL4065	atgatttctaagaaaaactttg	Fwd, for amplifying *apf1* (LAR_0410) gene
VPL4066	ttagtaccagccattagct	Rev, for amplifying *apf1* (LAR_0410) gene
VPL4067	gcagcagaaattgaaataaggtgatatttaatgatttctaagaaaaactttgctaaagta	LCR bridging oligo for ligating pMutL promoter sequence to *apf1* for insertion into pJP028 for complementation
VPL4068	gctcactggcaagctaatggctggtactaatgatctttgaaccaaaattagaaaaccaag	LCR bridging oligo for insertion of *apf1* into pJP028 for complementation
VPL4069	atgagaaattcgaatacaaataattg	Fwd, for amplifying *11993* (LAR_0044) gene
VPL4070	ttagttgtggcgcttctttg	Rev, for amplifying *11993* (LAR_0044) gene
VPL4071	gcagcagaaattgaaataaggtgatatttaatgagaaattcgaatacaaataattggcgt	LCR bridging oligo for ligating pMutL promoter sequence to *11993* for insertion into pJP028 for complementation
VPL4072	acttacagctcaaagaagcgccacaactaatgatctttgaaccaaaattagaaaaccaag	LCR bridging oligo for insertion of *11993* into pJP028 for complementation

^*a*^ oVPL: van Pijkeren Lab oligonucleotide identification number.

^*b*^ Fwd: forward; Rev: reverse; oligo: oligonucleotide. *cat*: chloramphenicol acetyltransferase; LAR_#### refer to closed reference genome *Limosilactobacillus reuteri* JCM1112; * indicates nonsense mutation.

### Integration of cat* in *L. reuteri* chromosome

The oligonucleotides used in this study can be found in Table 3. The P_HELP_::cat* cassette was amplified from pVPL3031 with oVPL283-284 and pVPL3038 (88) was amplified with oVPL271-272, followed by Gibson assembly (89) to yield pVPL3047. *L. reuteri* was transformed with 5  µg pVPL3047, and we screened by PCR for upstream and downstream single-crossover homologous recombination with oligonucleotides oVPL203-334-335 and oVPL202-334-335, respectively. Integration of cat* following double-crossover recombination was confirmed with oligonucleotide pair oVPL334-335 to yield *L. reuteri* VPL4011. Integration of oVPL283 by ssDNA recombineering (22) reverted the in-frame stop codon to its original DNA sequence to yield *L. reuteri* VPL4052, which served as the control for growth and adhesion experiments.

### Construction of *L. reuteri* putative adhesion protein mutants and barcoding

Putative adhesin mutants were generated by single-stranded DNA (ssDNA) recombineering as described previously (22). Briefly, VPL4011 harboring pVPL2032, which provides inducible expression of the phage recombinase RecT, was simultaneously transformed with 100 µg oVPL3848, a degenerative oligonucleotide, targeting cat* and 100 µg oligonucleotide targeting a putative adhesin (Table 4). To restore *cat*, oVPL3848 contains three adjacent randomized bases targeting the stop codon and three additional randomized bases targeting nearby wobble bases in codons encoding A138, S140, and S142. Incorporation of oVPL3848 in the chromosome modifies the stop codon and wobble bases creating a mixture of unique barcodes. Following selection on MRS supplemented with chloramphenicol, recombinant genotypes of genes encoding putative adhesins were identified by a mismatch amplification mutation assay (MAMA) PCR (90, 91). The integrity of recombinant genotypes was confirmed by Sanger sequencing. Each barcode was subsequently introduced in VPL4011 harboring pVPL2032 *via* recombineering, resulting in a group of control strains (Table 4).

**TABLE 4:** Recombineering and mutant screening oligonucleotides

Oligonucleotides^*a*^	Sequence (5’−3’)	Target/comment^*b*^	Locus^*c*^	Mutation(s)^*d*^
**VPL236**	**tcaaaccaccaggaccaagcgctgaaagacgacgcttTCTGCttaattcacctaatgggttggtttgatccatgaactgg**	Targets *rpoB*	LAR_1402	H488R
**VPL449**	**aaacgcgatccatgttggtgatataaatcatctgccctTGTCAagcatgatagtacaatggagaaaagaggattttgctcc**	Targets *srtA*	LAR_0227	K150*V151Q
VPL468	tcctaattcgcaaaataagcagagg	Fwd, starts 500 bp upstream of site mutated by oVPL449		
VPL469	aatggattacaaatacaggcaaaatcc	Rev, starts 500 bp downstream of site mutated by oVPL449		
VPL470	ttggtgatataaatcatctgccctTGTC	MAMA oligo that will form 500 bp amplicon when oVPL449 is incorporated		
**VPL1670**	**cgttaaaataggaaaacctttgcttaggtcaaatcgcaAGCTTtatccgaaaacagatttagtacctgttcctgtccgat**	Targets *thyA*	LAR_0739	Y38*Q39SM40L
VPL1671	gctatttcttagataaagtggctgac	Fwd, starts 500 bp upstream of site mutated by oVPL1670		
VPL1672	tttgcttaggtcaaatcgcaagctt	MAMA oligo that will form 500 bp amplicon when oVPL1670 is incorporated		
VPL1673	aaaattggaacatggtgtgacatgga	Rev, starts 500 bp downstream of site mutated by oVPL1670		
**VPL3694**	**acattttctgcattagttgcttgttgagcagatagcttTCACCggtaagcatcattttccttagcaacagctgagttgtaa**	Targets *11993*	LAR_0044	Q211RG212*
VPL3695	agttcgggcaactgctgatc	Fwd, starts 500 bp upstream of site mutated by oVPL3694		
VPL3696	gcttgttgagcagatagcttTCACC	MAMA oligo that will form 500 bp amplicon when oVPL3694		
VPL3697	taaccgcattgtaaaattcacggtagt	Rev, starts 500 bp downstream of site mutated by oVPL3694		
**VPL3763**	**catacccacgaatccaaattactgagatcccatacaaaTGATAggcggttccaactaattttacaatgacaatgcggaaat**	Targets *fbpA*	LAR_0878	N68*P69*
VPL3764	ggtgattaatactggctctggattttc	Fwd, starts 500 bp upstream of site mutated by oVPL3763		
VPL3766	gacaacatgaatattattagccgccg	Rev, starts 500 bp downstream of site mutated by oVPL3763		
VPL3836	tggaaccgccTATCA	MAMA oligo that will form 500 bp amplicon when oVPL3763		
**VPL3796**	**aacactatatccagttttacttaattcataagtatcatCCTATtttactttaacaacaacagcattactataattgcttcc**	Targets *cmbA*	LAR_0958	P282*N283D
VPL3797	tacaagcccttaaagtca	Fwd, starts 500 bp upstream of site mutated by oVPL3796		
VPL3798	ttgttgttaaagtaaaATAGG	MAMA oligo that will form 500 bp amplicon when oVPL3796		
VPL3799	atgttacctcatcagct	Rev, starts 500 bp downstream of site mutated by oVPL3796		
**VPL3802**	**ttgatatttggctaggtcagaccaatcagtagtcgtttATTACgtacttgcttcatccttattagtctggaccattggcgt**	Targets cyclic-phosphodiesterase (cidi)	LAR_0983	P58*Q59*
VPL3837	tggtagggaagtaatttcaatccc	Fwd, starts 500 bp upstream of site mutated by oVPL3802		
VPL3838	tcactggcaagtactgaatgttgg	Rev, starts 500 bp downstream of site mutated by oVPL3802		
VPL3839	gtcagaccaatcagtagtcgtttATTAC	MAMA oligo that will form 500 bp amplicon when oVPL3802		
**VPL3808**	**aatttttatacgcttgattcttagaagttaagtttcctCATCAataataagtaatataatcaagcattgatctttcataaa**	Targets *pilP*	LAR_0989	N162*Q163*
VPL3809	tctaacttttgaagtaattc	Fwd, starts 500 bp upstream of site mutated by oVPL3808		
VPL3810	gaagttaagtttcctCATCA	MAMA oligo that will form 500 bp amplicon when oVPL3808		
VPL3811	gactggccttttgtaatt	Rev, starts 500 bp downstream of site mutated by oVPL3808		
**VPL3814**	**aaagtgaagttacaattggtgtatttaaattttgtaatCATCAgtcattagttttaattacatttttatttctgttagtaa**	Targets *slpA*	LAR_1193	V102*Q103*
VPL3815	actatcagaacccgttag	Fwd, starts 500 bp upstream of site mutated by oVPL3814		
VPL3816	attaaaactaatgacTGATG	MAMA oligo that will form 500 bp amplicon when oVPL3814		
VPL3817	gaatacttgctgactagt	Rev, starts 500 bp downstream of site mutated by oVPL3814		
**VPL3850**	**ccaaccatcttttactggtgttgatatctctgttgactACTACgttactgagtttgctttttgatccgtggcttgttgagt**	Targets *apf1*	LAR_0410	D82E G83* E84*
VPL3851	ggattgacggtaatcattgtctac	Fwd, starts 500 bp upstream of site mutated by oVPL3850		
VPL3983	tgttgatatctctgttgactACTAC	MAMA oligo that will form 500 bp amplicon when oVPL3850		
VPL3984	ggcttatagccgatgtgca	Rev, starts 500 bp downstream of site mutated by oVPL3850		
**VPL3856**	**tgcattagctgcgttttgagcgttgtattcttgaatttACTACtcgctcttgatgattaacttttgaccaacgtaaatctt**	Targets autolysin (auto)	LAR_1284	P99* K100*
VPL3857	ggtgctgttacagcttagta	Fwd, starts 500 bp upstream of site mutated by oVPL3856		
VPL3858	gttaatcatcaagagcgaGTAGT	MAMA oligo that will form 500 bp amplicon when oVPL3856		
VPL3859	ctcgacctatacctgtcgaa	Rev, starts 500 bp downstream of site mutated by oVPL3856		
**VPL3939**	**agcgaatcccatttagttggtacaaagttagcttttaaTTATCtctttttagcaactgctttaccaagatctacttcaaag**	Targets *cnBp*	LAR_0284	M76RG77*
VPL3752	tccgaatgaattatctggcggac	Fwd, starts 500 bp upstream of site mutated by oVPL3939		
VPL3754	gctggatcttgttcactagaaacat	Rev, starts 500 bp downstream of site mutated by oVPL3939		
VPL3940	taaagcagttgctaaaaagaGATAA	MAMA oligo which will form 500 bp amplicon when oVPL3939		
VPL3993	AAACagatcttggtaaagcagttgctaaaaagatG	*cnBp* protospacer sequence		
VPL3994	AAAACatctttttagcaactgctttaccaagatct	*cnBp* protospacer sequence		
**VPL3848**	**gtttcccaaaacacctatacctgaaaatgcNttttcNNNNtcNattattccttggacttcatttactgggtttaacttaa**	Targets cat*. Incorporates random bases at stop codon and bases encoding A138, S140, and S142	N/A	
**VPL3996**	**gtttcccaaaacacctatacctgaaaatgcTttttcGACGtcTattattccttggacttcatttactgggtttaacttaa**	Δ*cmbA* barcode	N/A	
VPL3997	**gtttcccaaaacacctatacctgaaaatgcTttttcTTGCtcGattattccttggacttcatttactgggtttaacttaa**	Δautolysin barcode	N/A	
VPL3998	**gtttcccaaaacacctatacctgaaaatgcGttttcTATTtcTattattccttggacttcatttactgggtttaacttaa**	Δ*apf1* barcode	N/A	
VPL3999	**gtttcccaaaacacctatacctgaaaatgcCttttcGACAtcTattattccttggacttcatttactgggtttaacttaa**	Δ*11993* barcode	N/A	
VPL4000	**gtttcccaaaacacctatacctgaaaatgcTttttcCTTAtcCattattccttggacttcatttactgggtttaacttaa**	Δ*fbpA* barcode	N/A	
VPL4001	**gtttcccaaaacacctatacctgaaaatgcGttttcTATCtcTattattccttggacttcatttactgggtttaacttaa**	Δ*srtA* barcode	N/A	
VPL4002	**gtttcccaaaacacctatacctgaaaatgcTttttcAGCAtcTattattccttggacttcatttactgggtttaacttaa**	Δ*slpA* barcode	N/A	
VPL4003	**gtttcccaaaacacctatacctgaaaatgcTttttcAGTTtcAattattccttggacttcatttactgggtttaacttaa**	Δ*pilP* barcode	N/A	
VPL4005	**gtttcccaaaacacctatacctgaaaatgcTttttcGTGTtcTattattccttggacttcatttactgggtttaacttaa**	Δcidi barcode	N/A	

^*a*^ oVPL: van Pijkeren Lab oligonucleotide identification number. Bold indicates recombineering oligonucleotide; uppercase bases indicate mismatches with wild-type sequence. All recombineering oligonucleotides target the lagging strand.

^*b*^ Fwd: forward; Rev: reverse; oligo: oligonucleotide.

^*c*^ LAR_#### refer to the fully annotated and closed reference genome of *Limosilactobacillus reuteri* JCM1112 and can be found on https://www.ncbi.nlm.nih.gov.

^*d*^ * indicates nonsense mutation.

To compare the recombinant recovery frequency, we transformed strain VPL4011 simultaneously with oligonucleotides oVPL3848 and oVPL236. Transformed cells were plated on MRS agar supplemented with either chloramphenicol or rifampicin. Each colony on MRS agar plate supplemented with chloramphenicol was then patch plated onto MRS plate containing rifampicin.

### Bacterial survival of putative adhesion mutants following gastrointestinal transit

Fifty-eight 6-week-old male C57BL/6 J mice were purchased from The Jackson Laboratory (Bar Harbor, ME). Prior to the start of the experiment, the animals were allowed to adjust to the new environment for 1 week. The animals were individually housed in an environmentally controlled facility with a 12 h light and 12 h dark cycle. Food (standard chow; LabDiet, St. Louis, MO) and water were provided *ad libitum*. Two treatment groups were used. First treatment group contained five mice to test only single mutants, and the second treatment group consisted of eight mice to test WT mix and mutant mix. A mix of VPL4011 (*n* = 8) transformed with an oligonucleotide conferring each mutant barcode served as a control (WT mix). Mutant mix consisted of WT and all single mutants in 1:1:1:1:...1 ratio. Mice (*n*  =  5–8/treatment group) were gavaged for two consecutive days with 100 µL in PBS suspension containing ~10^9^ CFU/mL of chloramphenicol-resistant *L. reuteri* adhesion mutants and control strain VPL4052. Fresh fecal samples were collected 15, 27, and 39 h after the last oral administration and weighed. The fecal material was resuspended in PBS to 100 mg/mL and plated on MRS agar plates containing 5  µg/mL chloramphenicol. Cell viability counts were normalized per 10^8^ CFU administered *L. reuteri*.

### Inactivation of nine genes encoding putative adhesins in a single genetic background

*L. reuteri* VPL4018 is a derivative of *L. reuteri* VPL1014 in which the gene encoding sortase was inactivated (23). Strain VPL4018 was transformed with pVPL2032, which encodes RecT; this strain was subjected to ssDNA recombineering to generate a double mutant, followed by generating a triple mutant *etcetera* until total nine genes were inactivated to yield a nonuple mutant (Table 4). The mutations were achieved in the following order: (1) Δ*srtA*, (2) Δ*slpA*, (3) Δ*cmbA*, (4) Δ*auto*, (5) Δ*apf1*, (6) Δ*pilP,* (7) Δ*11993*, (8) Δ*fbpA*, and (9) Δ*cidi*.

### Construction of *L. reuteri* Δ*cnBp* single mutant

We were unable to identify a recombinant genotype for *cnBp* with single-stranded DNA recombineering. To increase efficiency, we employed CRISPR-Cas9-assisted recombineering (23). We first generated a plasmid encoding gRNA targeting *cnBp*. Briefly, pVPL3115 (23) was digested with Eco31I (Thermo Fisher Scientific followed by gel purification (Thermo Fisher Scientific, FERK0701). A pair of complementary oligonucleotides (oVPL3993–oVPL3994) identical to the 30 bp target region of *cnBp* was annealed to digested pVPL3115. DNA was mixed at a 1:1 molar ratio followed by overnight ligation, pellet paint precipitation and transformation in *L. lactis* MG1363. Insertion of the *cnBp* protospacer, yielding pCRISPR-*cnBp* was confirmed by sequence analysis. *L. reuteri* VPL3187 harboring pVPL3004 (23) and pVPL3016 (22, 23) was then co-transformed with oVPL3939 and pCRISPR-*cnBp* as described previously (23) to generate Δ*cnBp* (M76RG77*). Following genotype confirmation through PCR, Δ*cnBp* was passaged in MRS until Cm^-^, Em^-^, and Tet^-^ phenotypes were confirmed by restored antibiotic sensitivity. Plasmid loss was subsequently validated by PCR, and the subsequent strain VPL4379 (Δ*cnBp*) was confirmed by Sanger sequencing.

### Complementation of genes encoding adhesion proteins

For complementation, the target gene was cloned into a high-copy expression vector *via* ligase cycle reaction (LCR) (14). Briefly, we amplified the backbone of pJP028 (derived from pNZ8048) with primer pair oVPL1286–oVPL1408. We placed each gene under the control of the *L. reuteri* pMutL promoter, a promoter located upstream of the gene encoding MutL, which is involved in DNA repair (33, 92). We amplified pMutL with oVPL736 and oVPL737 using pSIP411:pMutL-ThyA as the template. Each gene encoding putative adhesion protein was amplified from their start to stop codons using the oligonucleotides listed in Table 3. First, *cmbA* amplified with oVPL4033 and oVPL4034 was ligated to pMutL and the pJP028 backbone with bridging oligonucleotides oVPL4035, oVPL4036, and oVPL4037. Next, 5 µL of the LCR mixture was directly transformed into *E. coli* EC1000 and plated on LB plates supplemented with 300 µg/mL erythromycin. Insertion of *pMutL* and *cmbA* into pJP028 was confirmed *via* PCR with oVPL329 and oVPL363, followed by Sanger sequencing. The resulting plasmid (pVPL31467) was amplified with oVPL737 and oVPL1286 to insert the remaining genes encoding putative adhesion proteins. Each complementation plasmid was then amplified with oVPL309 and oVPL310 to omit the *cat* cassette from the pJP028 backbone, which would otherwise interfere with the ability to distinguish strains *via* their barcodes. Finally, each complementation plasmid was electroporated into *L. reuteri* in which the corresponding gene was inactivated.

### Adhesion assay on HT-29 human colon cancer cells

*L. reuteri* wild type (VPL1014) and its derivative mutants were tested both individually and in the form of three types of mixtures (WT-mix, mutant mix, and complemented mix) for their competitive ability to adhere to the HT-29 (ATCC HTB-38). WT mixture consisted of VPL1014 and VPL4052, mutant mixture was obtained by mixing VPL1014 and each single adhesion mutant, and complemented mixture contained VPL31134 (empty vector control of *L. reuteri*) and complemented strain of each single adhesion mutant. To prepare the epithelial cells, the cells were seeded at 2 × 10^4^ cells/well (passages 4–9) and grown to 100% confluency in a 24-well plate (Biolite, Thermo ScientificTM, 12–556-006). Confluency was checked through microscopy to determine if cells form a monolayer with tight junctions between cells and display a typical apical brush border. For bacterial cell preparation, overnight (~16 h) cultures of all WT controls, single mutant strains and complemented strains were separately inoculated in fresh and pre-warmed MRS broth or MRS broth supplemented with erythromycin (5 µg/mL) at an OD_600_ = 0.1 and cultured up to OD_600_ = 1.0. One milliliter of each bacterial culture was harvested by centrifugation for 1 min at 21,130*×g*. The cell pellet was washed once in 1 mL PBS, centrifuged at 21,130×*g* for 1 min, and resuspended in 1 mL PBS. For each mixture, the cells of an individual strain were mixed in 1:1 ratio by adding 500 µL of PBS suspension of each strain. For the adhesion assay, the cell culture medium was removed, and the cells were washed with 1 mL of pre-warmed PBS. In a volume of 500 µL of bacterial mixture, ~5 × 10^6^ bacterial cells were added to the monolayer (MOI = 5:1). After 30 min of incubation at 37°C and 5% CO_2_, the cell layer was gently washed with PBS five times to remove non-adherent bacteria. After the final wash, the cells were lysed by adding ice-cold dH_2_O, and the cell layer was disrupted with a 1 mL pipette tip. The remaining adhered bacteria in the suspension were vigorously vortexed and serially diluted in PBS. Standard plate counts were used to enumerate both individual and mixture strains for pre-adhesion assay and only mixture strains for post-adhesion assay. Adhesion competition ratios were calculated as the ratio of the mutant or the complemented mutant (all chloramphenicol-resistant) to the wild-type strain as described previously (46). Six biological replicates were performed for each adhesion mutant and complemented mutant pair.

### Adhesion assay on human colon cancer enteroid monolayers

All single mutant strains, complemented strains, and barcoded controls were grown individually overnight (~16 h) in MRS broth supplemented with erythromycin (5 µg/mL) as needed. Overnight cultures of these strains were diluted to OD_600_ = 0.1 and grown to OD_600_ = 1.0 at which point 1 mL of cell suspension was centrifuged at 21,130×*g* for 1 min to harvest cell pellet. The cells were resuspended in 1 mL pre-warmed DMEM/F12 (Thermo Fisher), and cell concentration was quantified by plating serial dilutions on MRS and MRS supplemented with erythromycin (5 µg/mL) plates. Mixtures of ~1:1:1:1:…1 ratios of single mutant strains, complemented strains, or barcoded controls were prepared by mixing equal volumes of each strain in a single tube. The resulting mixtures were then diluted to achieve a final MOI of 5 and plated to quantify total cell concentration. Next, 250 µL of each mixture was added to six wells each of enteroid monolayers for three technical replicates of washed wells and unwashed control wells per group. After 1 h incubation at 37 °C in a humidified atmosphere of 5% CO_2_, the bacterial suspensions were carefully aspirated, and three wells of each mixture were washed three times with pre-warmed PBS. The remaining three wells per mixture serve as unwashed controls. After the last wash, enteroid cells were lysed by adding 1 mL (or 750 µL for unwashed wells) of ice-cold dH_2_O. The monolayers were then disrupted with a pipette tip to create a suspension of enteroid cells and bacteria before transferring the suspension to a 1.5 mL tube. After vortexing vigorously for 40 s, adhered bacterial cells were quantified by plating serial dilutions just as described by van Pijkeren et al. (46). The total DNA from the cell suspensions was then extracted by bead beating. Briefly, 300 µL of zirconia glass beads (BioSpec) and the cell suspensions were added to 2 mL microvials before loading onto a bead beater (BioSpec, Mini-Beadbeater-16) and beat for 3 min at 30-s intervals, with 30 s on ice in between beating. The cell lysate was harvested by centrifugation at (21,130×*g* for 1 min). DNA was extracted from the cell lysates with the Qiagen DNeasy Blood and Tissue Kit according to the manufacturer’s instructions. DNA samples were then prepared for NGS sequencing. In this experimental setup, the rifampicin-resistant control strain VPL4216 and the nonuple mutant were mixed in 1:1 ratio and plated for quantification prior to the addition to the monolayers (Fig. 5d, T_0_). The nonuple mutant and VPL4216 mixture were then added to enteroid monolayers to yield a total MOI of 5:1 or 30:1, and co-incubated for Nonuple mutant was also competed with rifampicin-resistant VPL4216 [*L. reuteri::rpoB*(H488R)]. Construction of VPL4216 was described previously (22, 34). The procedure was identical to the pooled adhesion competition assay, where the adhesive ability of all single mutants was compared in a single attempt, except that cells were plated on MRS with or without rifampicin supplementation (25 µg/mL) to differentiate between nonuple and VPL4126. The resulting ratio of adhered VPL4126 to nonuple cells was determined by comparing cell counts on MRS plates supplemented with rifampicin (VPL4126 count) and total cells on MRS plates. Nonuple cell counts were calculated by subtracting the rifampicin-resistant CFU from the total CFU count. Experiments were performed with three biological replicates with three technical replicates each.

### Library preparation of adhesion competition DNA samples

For NGS sequencing, DNA samples were prepared by PCR enrichment (Roche, KAPA HiFi) with oligonucleotides that add Illumina adapters (oVPL4155, oVPL4156, oVPL4157, and oVPL4158). Oligonucleotides oVPL4157 and oVPL4158 have six N’s in between the annealing sequence and the Illumina adapters to add sequence diversity at the ends of each amplicon. After confirming the absence of primer–dimers *via* gel electrophoresis, enriched samples were purified with a GeneJet purification kit (ThermoFisher, FERK0701). Next, 10 µL of each enriched sample was used as template for index PCR with KAPA HiFi (eight cycles). Oligonucleotides used for sample enrichment and indexing are listed in Table 5, and were designed to distinguish samples from all biological and technical replicates. Indexed samples were then purified and quantified (Qubit fluorometric quantification; Life Technologies).

**TABLE 5:** Oligonucleotides used for library preparation

Oligonucleotides^*a*^	Sequence (5’−3’)	Target/comment^*b*^
VPL4155	acactctttccctacacgacgctcttccgatctcctgctgtaataatgggtagaagg	Fwd, internal to *cat* gene with Illumina adapter
VPL4156	gtgactggagttcagacgtgtgctcttccgatcttggactcctgtaaagaatgacttca	Rev, internal to *cat* gene with Illumina adapter
VPL4157	acactctttccctacacgacgctcttccgatctNNNNNNcctgctgtaataatgggtagaagg	Fwd, internal to *cat* gene with Illumina adapter; “N’s” indicate degenerate nucleotides
VPL4158	gtgactggagttcagacgtgtgctcttccgatctNNNNNNtggactcctgtaaagaatgacttca	Rev, internal to *cat* gene with Illumina adapter; “N’s” indicate degenerate nucleotides
VPL4210	AATGATACGGCGACCACCGAGATCTACACcggaagaaACACTCTTTCCCTACACGACGCT	Fwd, stem and index i5 for illumina sequencing_1
VPL4211	CAAGCAGAAGACGGCATACGAGATttcttccgGTGACTGGAGTTCAGACGTGTGC	Rev, stem and index i7 for illumina sequencing_1
VPL4212	AATGATACGGCGACCACCGAGATCTACACgacaccaaACACTCTTTCCCTACACGACGCT	Fwd, stem and index i5 for illumina sequencing_2
VPL4213	CAAGCAGAAGACGGCATACGAGATttggtgtcGTGACTGGAGTTCAGACGTGTGC	Rev, stem and index i7 for illumina sequencing_2
VPL4214	AATGATACGGCGACCACCGAGATCTACACacaactggACACTCTTTCCCTACACGACGCT	Fwd, stem and index i5 for illumina sequencing_3
VPL4215	CAAGCAGAAGACGGCATACGAGATccagttgtGTGACTGGAGTTCAGACGTGTGC	Rev, stem and index i7 for illumina sequencing_3
VPL4216	AATGATACGGCGACCACCGAGATCTACACcggtactaACACTCTTTCCCTACACGACGCT	Fwd, stem and index i5 for illumina sequencing_4
VPL4217	CAAGCAGAAGACGGCATACGAGATtagtaccgGTGACTGGAGTTCAGACGTGTGC	Rev, stem and index i7 for illumina sequencing_4
VPL4218	AATGATACGGCGACCACCGAGATCTACACactcacacACACTCTTTCCCTACACGACGCT	Fwd, stem and index i5 for illumina sequencing_5
VPL4219	CAAGCAGAAGACGGCATACGAGATgtgtgagtGTGACTGGAGTTCAGACGTGTGC	Rev, stem and index i7 for illumina sequencing_5
VPL4220	AATGATACGGCGACCACCGAGATCTACACctagacgaACACTCTTTCCCTACACGACGCT	Fwd, stem and index i5 for illumina sequencing_6
VPL4221	CAAGCAGAAGACGGCATACGAGATtcgtctagGTGACTGGAGTTCAGACGTGTGC	Rev, stem and index i7 for illumina sequencing_6
VPL4222	AATGATACGGCGACCACCGAGATCTACACataggtcgACACTCTTTCCCTACACGACGCT	Fwd, stem and index i5 for illumina sequencing_7
VPL4223	CAAGCAGAAGACGGCATACGAGATcgacctatGTGACTGGAGTTCAGACGTGTGC	Rev, stem and index i7 for illumina sequencing_7
VPL4224	AATGATACGGCGACCACCGAGATCTACACtagcagcaACACTCTTTCCCTACACGACGCT	Fwd, stem and index i5 for illumina sequencing_8
VPL4225	CAAGCAGAAGACGGCATACGAGATtgctgctaGTGACTGGAGTTCAGACGTGTGC	Rev, stem and index i7 for illumina sequencing_8

^*a*^ oVPL: van Pijkeren Lab oligonucleotide identification number.

^*b*^ Fwd: forward; Rev: reverse.

### Targeted sequence analysis from competition experiment on human enteroid monolayers

Sequencing was performed at the University of Wisconsin-Madison Biotechnology Center. Quality and quantity of the finished libraries were analyzed using Agilent Tapestation and Quantus Qubit dsDNA assay. The samples were diluted to 2 nM before sequencing. Paired-end, 150 bp sequencing was performed using the Illumina MiSeq Sequencer and a MiSeq 300 bp (v2) sequencing cartridge. Quality control images were analyzed using MultiQC v1.dev0. Paired-end Illumina sequencing reads were merged and filtered with PEAR (Paired-End reaD mergeR) using default settings (93). Phred quality scores (Q scores) were used to compute the total number of expected errors (E) for each merged read, and reads exceeding an Emax of 1 were removed. The ratios of strains before and after the adhesion assay on human colon cancer enteroid monolayers were determined by the number of filtered reads that matched each barcode compared with the total filtered reads within each sample. Reads that corresponded to barcodes not used in this study, which altogether constituted less than 4.1% of the total reads in each sample, were excluded from calculations.

### Growth and phage production of nonuple mutant

To determine growth and phage production by the nonuple mutant, we treated cells with mitomycin C following the protocol of Alexander *et al*. (33). Briefly, overnight (∼16 h) cultures were diluted to an OD600 of 0.1, and at an OD600 of 0.3, mitomycin C was added (0.5  µg/mL; Sigma-Aldrich). At the endpoint of the growth analysis (T_8_), the supernatant of each culture was harvested to determine the number of CFU and PFU per milliliter. For phage quantification, the cells were centrifuged (21,130×*g* for 1 min), and the supernatants were filter sterilized (pore size, 0.22  µm; Millipore). Lytic host suspension [*L. reuteri* LRΔΦ1ΔΦ2Δ*attB1ΔattB2* (VPL4090)] and phage samples (200 µL each) were equally mixed in a 15 mL conical tube, and the mixture was incubated at 37°C for 1  h. Then, 3  mL of 0.2% (wt/vol) agarose harboring 10  mM CaCl2 was added, which was gently inverted three times, and the mixture was poured onto MRS agar supplemented with 10  mM CaCl2, followed by 15 h of incubation at 37°C.

### Persistence of nonuple mutant vs WT in mouse model

Twelve 8-week-old male B6 mice were purchased from Jackson Laboratory (Bar Harbor, ME) and adapted for 1 week to the new environment before starting the experiment. The animals were housed at an environmentally controlled facility with a 12 h light and dark cycle. Global rodent diet (Teklad) and water were provided *ad libitum*. Mice (*n* = 6/group) received 100 µL nonuple or WT (10^9^ CFU) by oral gavage. After the second administration of each strain, we tracked daily the bacterial concentration in mouse feces by plating on MRS agar containing vancomycin (200 µg/mL) for 7 days. The clearance rate of bacteria from GI tract was determined as the absolute value from the slope of linear regression curve for the bacterial concentration [Log (CFU/100 mg)] over days post-oral gavage (0 to 5 days post-oral gavage), as shown in Fig. 5e.

### Therapeutic efficacy of nonuple mutant vs WT in mouse model

Adult 15 female C57BL/6NTac mice (Taconic Biosciences, Inc., Germantown, NY, USA) and C57BL/6 mice (8–10 weeks old) with knock-in allele-Lgr5-EGFP-IRES creERTZ (Lgr5 +GFP + ) were given deionized water and standard laboratory chow, and four mice were kept together in one cage. Mouse irradiation was performed just as described by Hamade *et al*. (37). Briefly, mice received 13.35 Gy partial body irradiation (PBI) using a Varian Trilogy linear accelerator (Palo Alto, CA, USA). The irradiation field was a 20 × 20 cm field with the mice placed in the irradiation field, except one leg that was placed outside of the irradiation field. This setup of irradiation preserved 5% of the bone marrow from irradiation. The mice were irradiated using 6 mV photons at 600 monitor units per minute. The mice were irradiated at a distance of 100 cm source-to-surface distance (SSD), which is a distance from the source of irradiation to the surface of the patient or radiation target. WT-IFN-β and nonuple-IFN-β were constructed by using previously published protocol (37). Briefly, single-stranded DNA recombineering was used to inactivate thyA in *L. reuteri* VPL1014 and nonuple mutant VPL4366. To replace chloramphenicol resistance gene with thyA gene in the WT and nonuple, blunt-end ligation (T4 DNA ligase, Thermo Fisher Scientific, Waltham, MA, USA) was used. Both strains were transformed through electroporation to construct WT-IFN-β and nonuple-IFN-β. Previously constructed vector pCtl-thyA placed in WT and nonuple mutant were used as empty vector controls. We used *E. coli* EC1000 as an intermediate cloning host. Mice were gavaged with 200 µL suspensions containing 100 µL saline and 100 µL of either 10^9^ WT-IFN-β or 10^9^ nonuple-IFN-β. Survival of animals was tracked for 30 days. After irradiation, the mice were sacrificed, and tissue collection was done following the protocols of Hamade *et al*. (37).

### Statistics

Data representation was performed using DataGraph (version 4.3) software (Visual Data Tools, Inc., Chapel Hill, NC, USA) and GraphPad Prism 9 software package (GraphPad Software Inc.). Statistical comparisons were performed using a paired t test, one-way analysis of variance, and Tukey’s honestly significant difference test (HSD) (JMP Pro software, version 14.0.0). Three biological replicates were performed for all *in vitro* studies. All samples were included in the analyses, and experiments were performed without blinding. For mouse survival experiments, Kaplan–Meier survival curves were plotted for data collection of each group. The comparison between each treated group and control was performed by using the two-sided log-rank test. *P* < 0.05 was considered as significant for statistical analyses and *P* values were not adjusted for multiple comparisons.

## Data Availability

The data that support the findings of this study are available from the corresponding author upon reasonable request.

## SUPPLEMENTAL MATERIAL

Supplemental material aem.00312-24-s0001.docx Figures S1 to S3.

ASM does not own the copyrights to Supplemental Material that may be linked to, or accessed through, an article. The authors have granted ASM a non-exclusive, world-wide license to publish the Supplemental Material files. Please contact the corresponding author directly for reuse.
