# Supplementary material for: Development of a Limosilactobacillus reuteri therapeutic delivery platform with reduced colonization potential
Source: Appl Environ Microbiol. 2024 Oct 31;90(11):e00312-24. doi: 10.1128/aem.00312-24 (PMC11577788; doi:10.1128/aem.00312-24)
Supplement: Supplemental material — Figures S1 to S3. [file aem.00312-24-s0001.docx]

**SUPPLEMENTARY MATERIAL**

**Development of a *Limosilactobacillus reuteri* therapeutic delivery platform with reduced colonization potential**

**Running Title:** Novel reduced colonization potential strategy for engineered microbes

**Laura M Alexander^1^, Saima Khalid^1^, Gina M Gallego-Lopez^2,6^, Theresa J Astmann^1^, Jee-Hwan Oh^1^, Mark Heggen^1^, Phil Huss^3^, Renee Fisher^4^, Amitava Mukherjee^4^, Srivatsan Raman^3^, In Young Choi^1^, Morgan N Smith^1^, Claude J Rogers^5^, Michael W Epperly^4^, Laura J Knoll^2^, Joel S Greenberger^4^, Jan-Peter van Pijkeren^1*^**

1 Department of Food Science, University of Wisconsin-Madison, Madison, WI, 53706, USA

2 Department of Medical Microbiology and Immunology, University of Wisconsin-Madison, Madison, WI, 53706, USA

3 Department of Biochemistry, University of Wisconsin-Madison, Madison, WI, 53706, USA

4 Department of Radiation Oncology, UPMC Hillman Cancer Center, Pittsburgh, PA 15232, USA.

5 ChromoLogic, LLC, Monrovia, CA 91016, USA

6 Morgridge Institute for Research, Madison, WI, 53715, USA.

*Correspondence:

Phone: +1 608 890 2640

Fax: +1 608 262 6872

Email: [vanpijkeren@wisc.edu](mailto:vanpijkeren@wisc.edu)


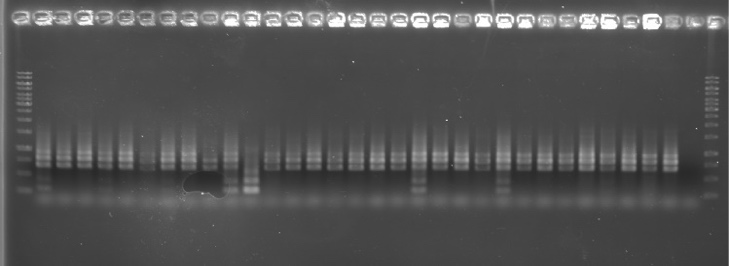

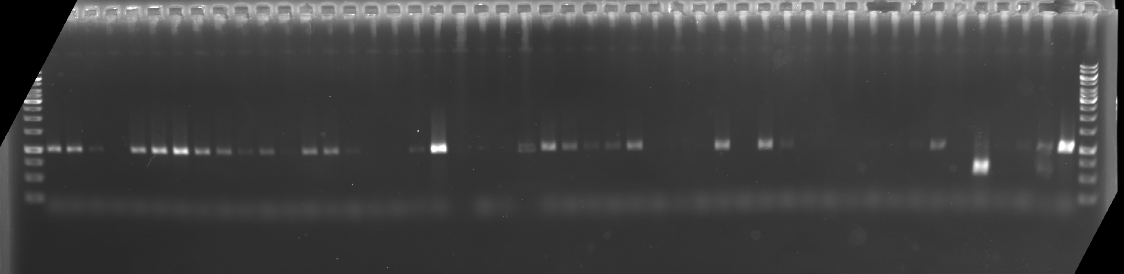


**c)** Δ*srtA*

*

*

(+)


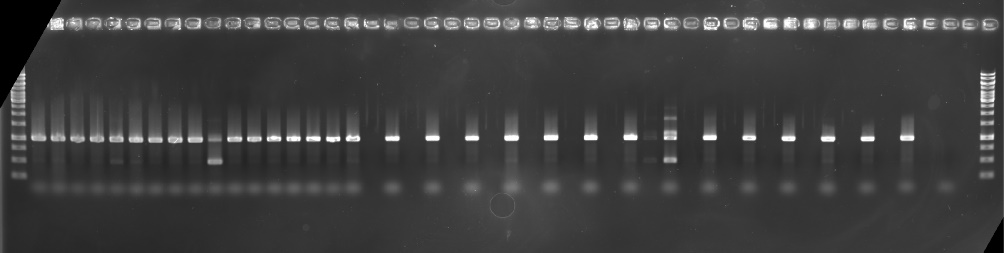


*

(+)

**b)** Δ*11993*

*

*


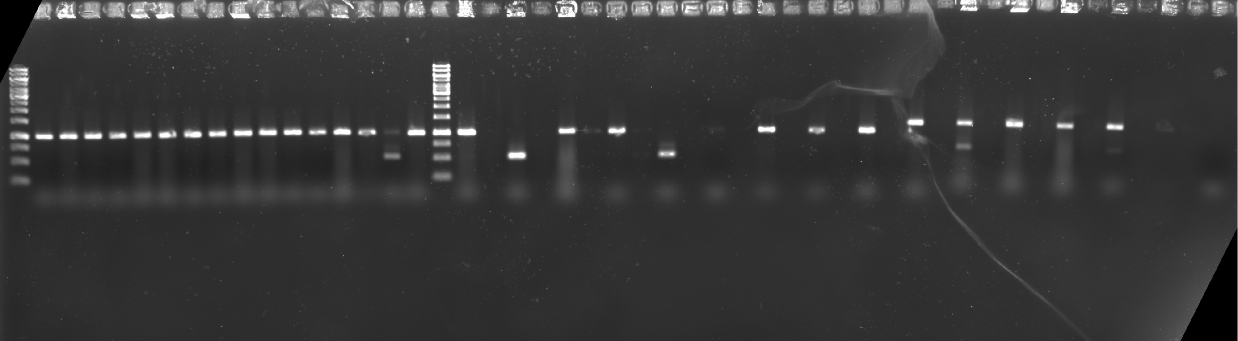


**d)** Δ*slpA*

*

*

*

*

(+)

(-)

**i)** Δ*cmbA*

**j)** Δ*slpA*


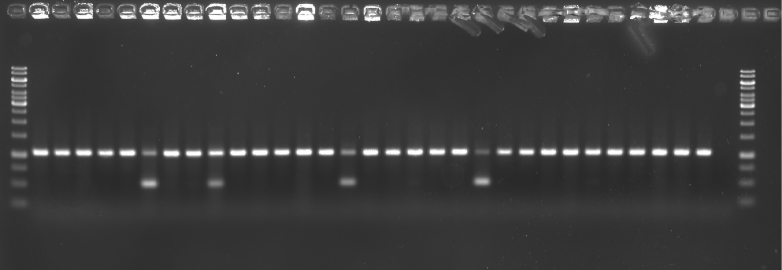


**g)** Δ*cidi*

*
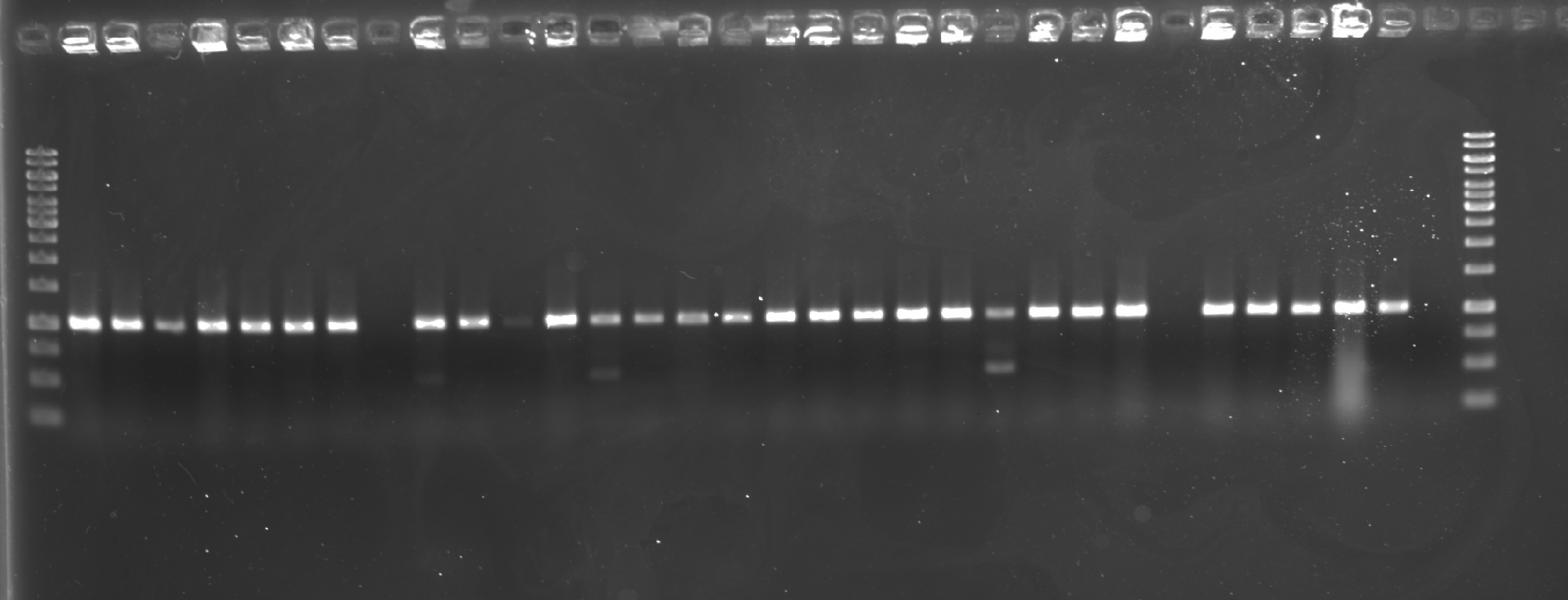


*

*

*

(+)

(-)


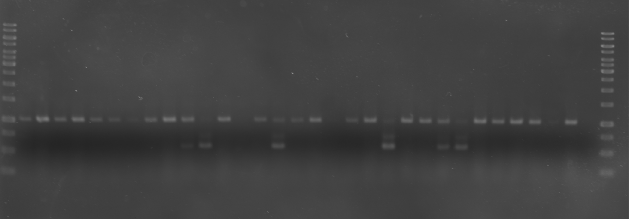


*

*

*

*

*

*

(+)

(-)


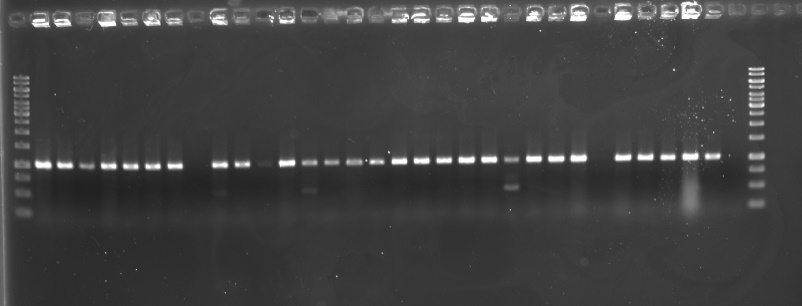


*
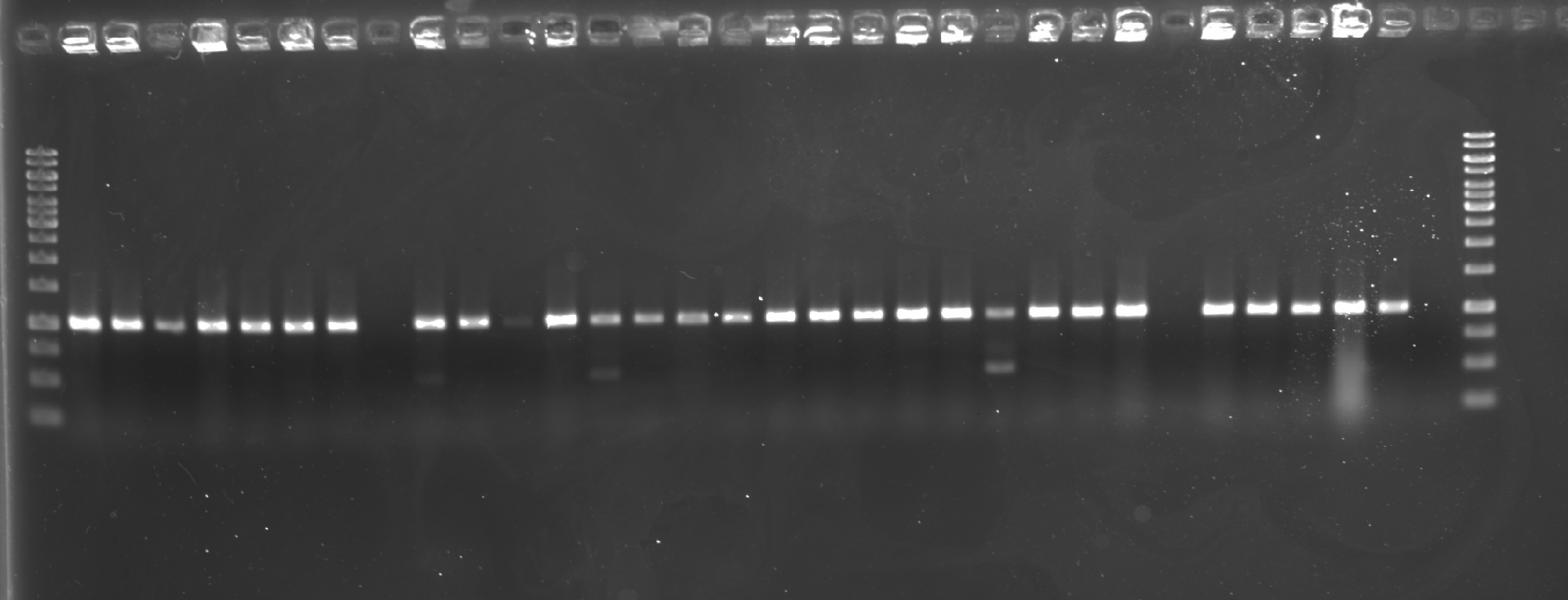


*
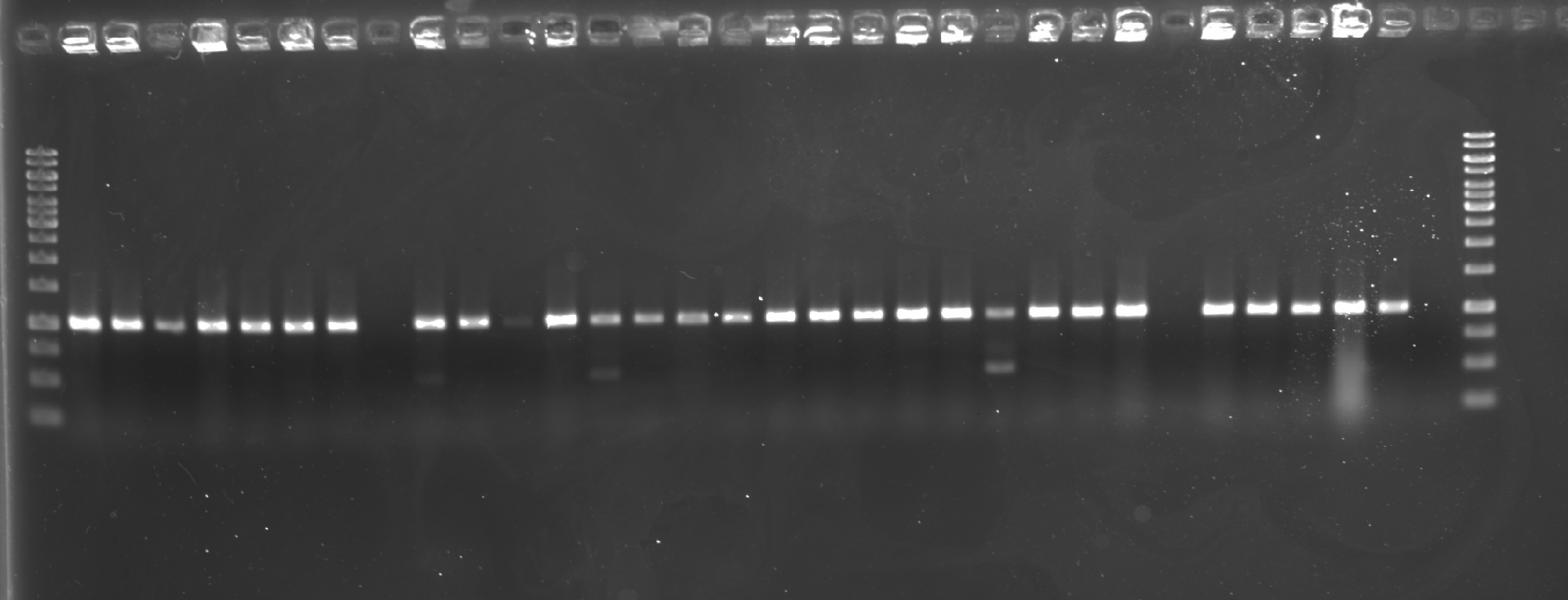


*
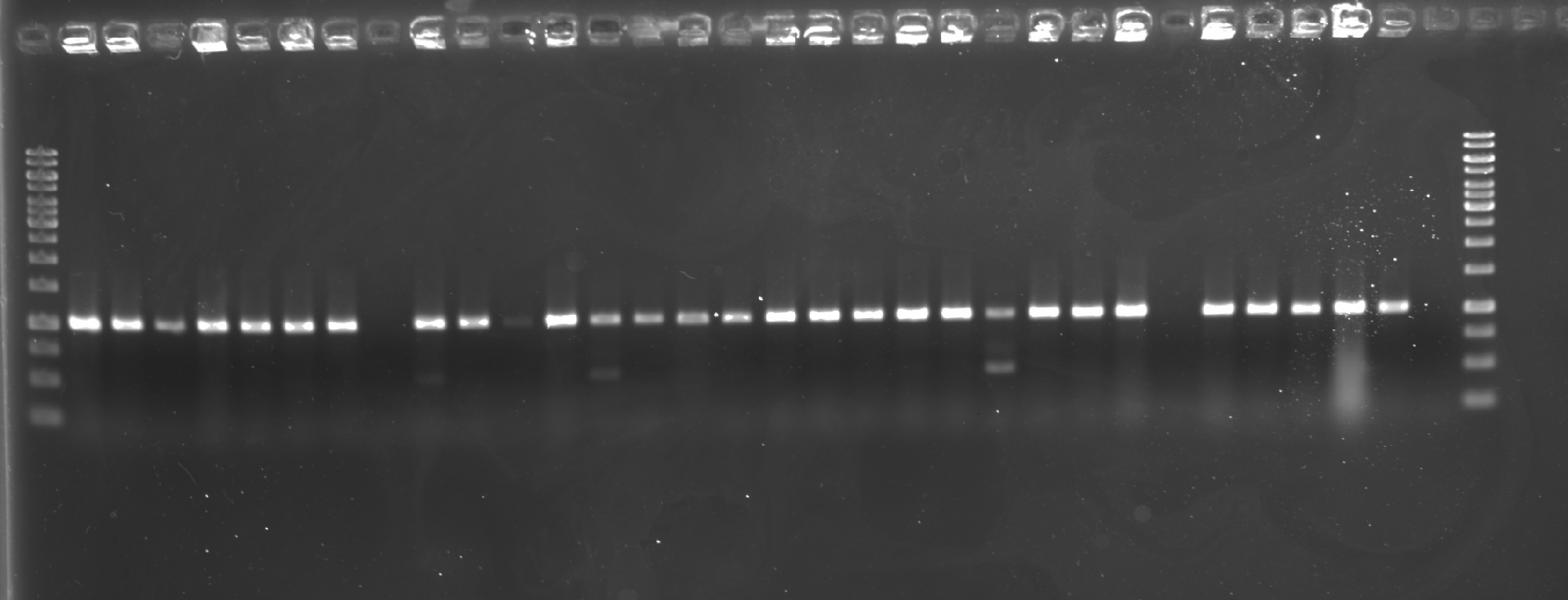


(+)

(-)

**h)** Δ*pilP*


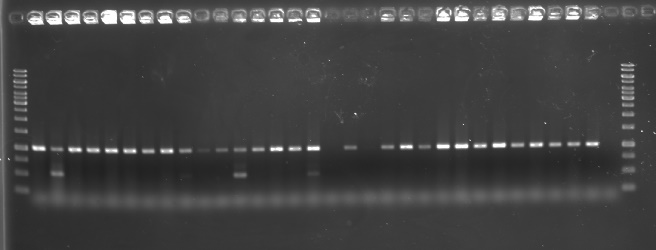


*

*

*

(+)

(-)


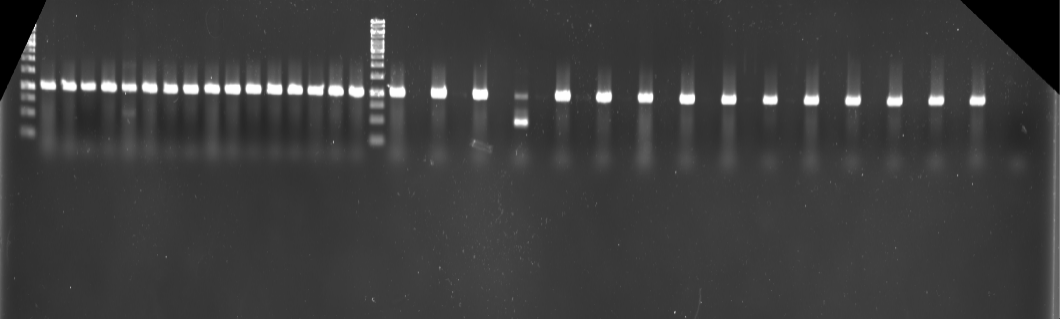


**a)** Δ*fbpA*

*

*

**e)** Δ*apf1*

*

*

*

*

(+)

(-)


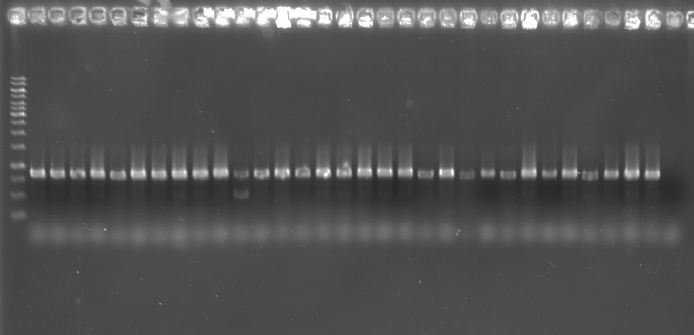


**f)** Δ*auto*

(+)

(-)

(-)

(-)

(+)

(-)

*

**Fig. S1.** DNA gel images of MAMA PCR screens for each adhesin mutant. **a)** Δ*fbpA*, **b)** Δ*11993*, **c)** Δ*srtA*, **d)** Δ*slpA*, **e)** Δ*apf1*, **f)** Δ*auto*, **g)** Δ*cidi*, **h)** Δ*pilP*, **i)** Δ*cmbA*, **j)** Δ*slpA*. Wild-type genotypes are represented by the 1 kb bands, while mutant genotypes are represented by the 0.5 kb bands (marked by red asterisks). Screening oligonucleotides are listed in Table 4. Thirty potential transformants were screened for each mutant. Following the initial screen, colonies that resulted in a 0.5 kb band were colony-purified and rescreened for pure genotype. Positive ((+), wild-type *L. reuteri*) and negative ((-), no template) PCR controls are indicated.

**Fig. S2.** Expression levels of the genes in *L. reuteri* VPL1014 WT analyzed by RNA-seq. *L. reuteri* VPL1014 WT was grown in modified MRS supplemented with 100 mM Glucose at 37°C until mid-log phase. RNA was extracted from the cells using the standard TRIzol method and subjected to RNA-seq analysis. Raw counts generated by RNA-seq by Expectation-Maximization (RSEM, v1.2.31) were then normalized using EdgeR (v3.42.4). Inter-sample normalization and intra-sample normalization were conducted with trimmed mean of M-values (TMM) and counts per million (CPM), respectively. GAPDH, a housekeeping gene is used as a reference gene. The results shown are averages from four independent experiments with ± standard error of the mean.


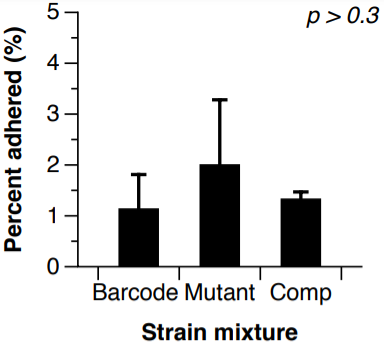


**Fig. S3.** Recovery of cells following adhesion assay competition presented as percent of the total mixed cells that adhered compared to the total amount of cells added to the enteroid monolayers. The results shown are averages from three independent experiments with three technical replicates each, ± standard error of the mean, *p* > 0.3.
